# Effects of Ischemia-Reperfusion on Tubular Cell Membrane Transporters and Consequences in Kidney Transplantation

**DOI:** 10.3390/jcm9082610

**Published:** 2020-08-12

**Authors:** Quentin Faucher, Hugo Alarcan, Pierre Marquet, Chantal Barin-Le Guellec

**Affiliations:** 1 IPPRITT UMR1248, Université de Limoges, INSERM, F-87042 Limoges, France; quentin.faucher@unilim.fr (Q.F.); hugo.alarcan@unilim.fr (H.A.); pierre.marquet@unilim.fr (P.M.); 2 Department of Pharmacology and Toxicology, CHU Limoges, F-87042 Limoges, France; 3 Laboratory of Biochemistry and Molecular Biology, CHU Tours, F-37000 Tours, France; 4 Department of Pharmacology, Université de Tours, F-37044 Tours, France

**Keywords:** ischemia/reperfusion injury, kidney transplantation, renal tubular transporters, drug transporters, toxin elimination

## Abstract

Ischemia-reperfusion (IR)-induced acute kidney injury (IRI) is an inevitable event in kidney transplantation. It is a complex pathophysiological process associated with numerous structural and metabolic changes that have a profound influence on the early and the late function of the transplanted kidney. Proximal tubular cells are particularly sensitive to IRI. These cells are involved in renal and whole-body homeostasis, detoxification processes and drugs elimination by a transporter-dependent, transcellular transport system involving Solute Carriers (SLCs) and ATP Binding Cassettes (ABCs) transporters. Numerous studies conducted mainly in animal models suggested that IRI causes decreased expression and activity of some major tubular transporters. This could favor uremic toxins accumulation and renal metabolic alterations or impact the pharmacokinetic/toxicity of drugs used in transplantation. It is of particular importance to understand the underlying mechanisms and effects of IR on tubular transporters in order to improve the mechanistic understanding of IRI pathophysiology, identify biomarkers of graft function or promote the design and development of novel and effective therapies. Modulation of transporters’ activity could thus be a new therapeutic opportunity to attenuate kidney injury during IR.

## 1. Introduction

For patients with end-stage renal disease, transplantation is a treatment of choice, providing a much better quality of life. To overcome the gap between a growing need for organs and the limited number of living or brain-dead donors, most centers are increasingly using sub-optimal donors, i.e., presenting with circulatory death or other expanded donation criteria. However, kidneys retrieved from such donors are more prone to Ischemia-Reperfusion (IR)-Induced acute kidney injury (IRI), Delayed Graft Function (DGF), and generally have shorter graft survival [1]. In the context of solid organ transplantation, IRI is strongly correlated to morbidity. Tissue and cellular hypoxia begin as soon as the organ is removed and the hypoxic phenomenon extends during graft storage in the preservation fluid. Graft lesions then increase after transplantation, secondary to revascularization. IRI is a complex pathophysiological process, associated with numerous structural and metabolic changes, arising from a complicated interaction between renal hemodynamics, inflammatory cytokines, endothelial and tubular cell injuries. These processes in turn favor short and potentially long-term graft failure [2,3,4,5,6,7,8].

The proximal tubule is more sensitive to IRI than other kidney structures. Proximal tubular cells express many multispecific transporters, belonging to either the SoLute Carrier (SLC) or ATP-Binding Cassette (ABC) families. They are involved in the reabsorption and secretion of various endogenous and exogenous compounds, according to their locations, i.e., on the basolateral (blood) or apical (urine) sides. These substrates comprise electrolytes (e.g., Na^+^, Cl^−^, K^+^, Ca^2+^), glucose, amino acids, several important anions (e.g., phosphate and citrate), uremic toxins (e.g., *p*-cresol, indoxyl sulfate, hippuric acid) as well as xenobiotics, some of which are used during the post-transplantation period (e.g., immunosuppressants, antibiotics, antiviral drugs). Owing to the bi-directional exchange they allow, proximal tubular cells are involved in renal and whole-body homeostasis, detoxification processes and xenobiotic clearance [9,10,11]. The effects of IR on the metabolism and structure of proximal tubular cells have been widely studied [12,13], but its effects on expression and activity of membrane transporters is less known and has not been reviewed so far. This article reviews current knowledge about the impact of IR on the expression and activity of SLC and ABC renal tubular cell membrane transporters. Its ambition is also to help to figure out new therapeutic approaches to prevent or reduce IRI-dependent alterations of transporter’s activity.

## 2. Coordinated and Bidirectional Transcellular Transport in Proximal Tubular Cells

**Figure 1:**
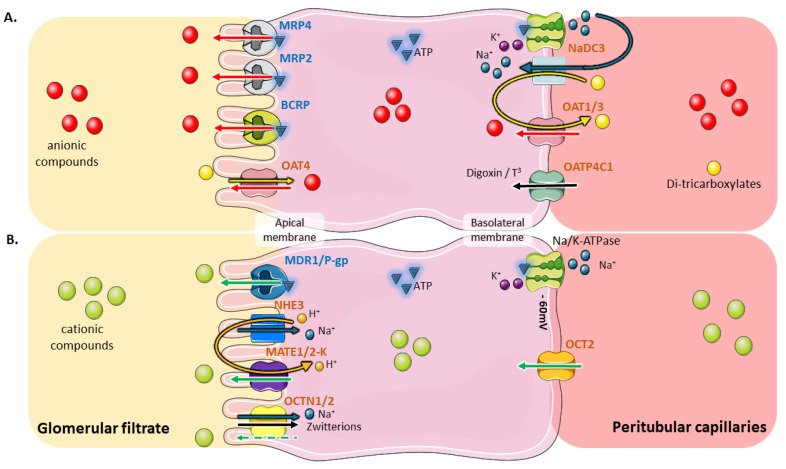
Bidirectional transporter-dependent transfer of anionic (**A**) or cationic (**B**) compounds within proximal tubular cells. (**A**) The elimination of organic anions (OA^−^) mainly depends on a coordinated transfer mediated by OAT1/3 (*SLC22A6* and *SLC22A8*) and MRP2/4 (*ABCC2* and *ABCC4*). The transport of OA^−^ at the basolateral side of tubular cells by OATs uses a tertiary active transport system. The Na^+^/K^+^-ATPase pumps sodium out of the cell. This creates the gradient by which Na^+^/dicarboxylate cotransporters (NaDC3/*SLC13A3*) drive the uptake of Na^+^ and α-ketoglutarate and other dicarboxylates into the cell. High intracellular concentration of dicarboxylates produces the outward driving force enabling influx of organic substrates by OATs exchangers. This antiport transfer results in the entry of endogenous OA^−^ or xenobiotics into the tubular cell. A member of the OATP family, OATP4C1 (*SLCO4C1*), also contributes to the basolateral entry of anionic molecules including cAMP, digoxin, ouabain, and thyroid hormones (T^3^) [16,17]. The OA^−^ that have reached the intracellular compartment are then excreted at the apical pole through a ATP-dependent primary active transport depending mainly on MRP2, MRP4 and BCRP (*ABCG2*) [14,16,18]. At the apical membrane, OAT4 (*SLC22A11*) promotes a bidirectional transport which allows the reabsorption of some OA^−^ including sulfate conjugates against the efflux of dicarboxylates such as α-ketoglutarate [19]. (**B**) Organic cations (OC^+^) pass through the basolateral membrane via an electrogenic uniport transport, driven by the negative internal potential created by the Na^+^/K^+^-ATPase. This transport mostly involves the OCT2 member of the SLC family (*SLC22A2*) [20]. At the apical membrane, MATE1 (*SLC47A1*) and MATE2-K (*SLC47A2*), coupled with Na^+^/H^+^ exchangers like NHE3 (*SLC9A3*), mediate the secretion of OC^+^: the protons released into the tubular lumen are taken up by OC^+^/H^+^ exchangers, promoting an input of H^+^ which is counterbalanced by an output of organic cations [15]. Other transporters of the SLC family are expressed at the apical membrane, including OCTN1 (*SLC22A4*) and OCTN2 (*SLC22A5*), which ensure the Na^+^-dependent reabsorption of zwitterions and facilitated secretion of OC^+^ [21]. MDR1/P-gp (*ABCB1*), an ABC family transporter located at the apical side, is also involved in the removal of organic cations and xenobiotics [9]. Artwork was designed using Servier Medical Art by Servier licensed under CC BY 3.0 (https://smart.servier.com).

Proximal tubular cells ensure trans-epithelial exchanges by means of their singular cyto-architecture and coordinated transport system. These polarized cells possess a basolateral membrane, in relation to systemic circulation, and an apical membrane made of elongated microvilli, in contact with the glomerular filtrate. Several transmembrane transporters, which mainly belong to the SLC and the ABC families, ensure coordinated movements of their substrates across tubular cells (Figure 1) [9,14,15].

The SLC superfamily includes a large number of polyspecific transporters, some of which are intensively expressed at the renal level. Among the most expressed ones, are organic cations facilitative uniporters (OCTs), organic anion/dicarboxylate exchangers (OATs), Na^+^/zwitterion cotransporters (OCTNs), uric acid exchanger (URAT1), Na^+^/phosphate (NaPi-IIa/NaPi-IIc) or Na^+^/dicarboxylate (NaDC3) cotransporters, sodium-independent organic anion transporters (OATPs) and multi-drug and toxin extrusion transporters (MATEs) [22,23,24]. ABC transporters, which promote ATPase-coupled unidirectional transport of their substrates, are also widely expressed at the apical membrane of tubular cells. The most expressed tubular ABC transporters are multidrug resistance protein 1 (P-gp, MDR1), multidrug resistance-associated proteins 2,4 (MRP2,4) and breast cancer resistance protein (BCRP) [14,25].

## 3. Role of Tubular Transporters in Maintaining Renal Cells’ Equilibrium, Tissue Homeostasis, Detoxification Processes and Drug Elimination

**Figure 2:**
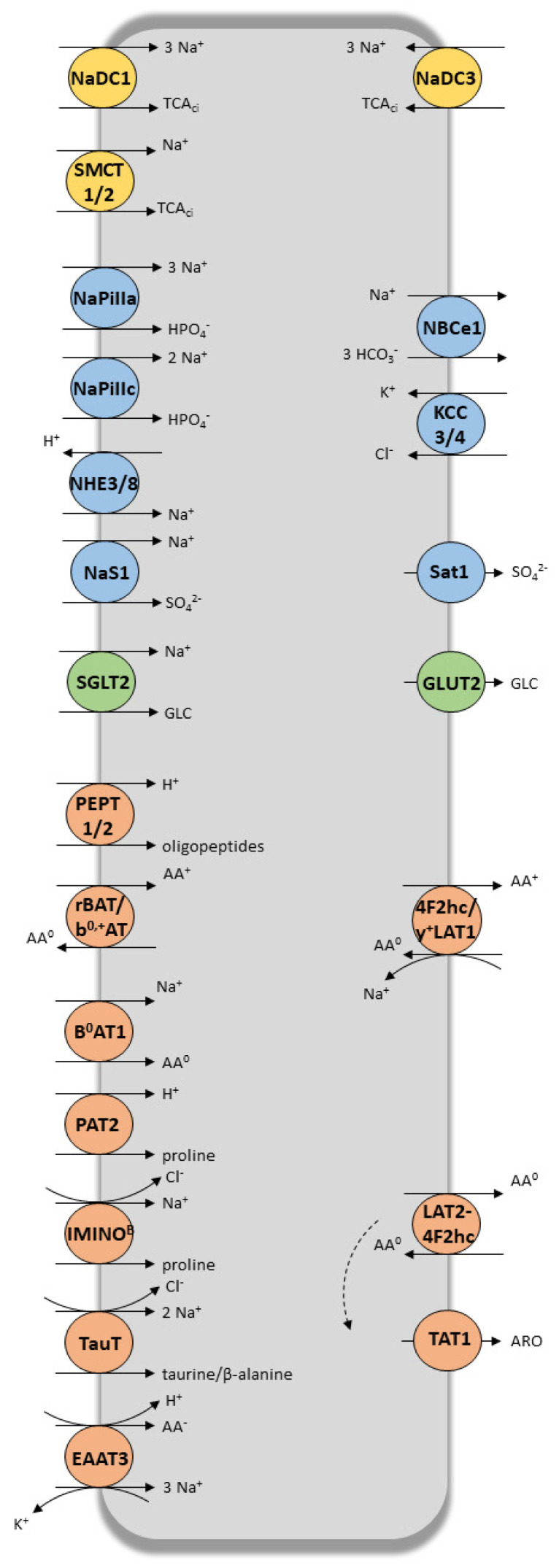
TCA cycle intermediates (yellow), electrolytes (blue), glucose (green) and peptides (orange) SLCs super-family transporters expressed at apical (left) and basolateral (right) membrane of tubular proximal cells. TCA_ci_, TCA cycle intermediates; Ur, urate; GLC, glucose; AA^−^, anionic amino acids; AA^0^, neutral amino acids; AA^+^, dibasic amino acids; ARO, aromatic amino acids. Artwork was designed using Servier Medical Art by Servier licensed under CC BY 3.0 (https://smart.servier.com).

Following glomerular filtration, numerous solutes (e.g., amino acids, glucose, potassium, phosphate, bicarbonate, low-molecular-weight proteins, tricarboxylic acid (TCA) intermediates) are reabsorbed in the proximal tubule to prevent excessive losses of vital metabolites. Tubular cells are also in charge of the urinary elimination of various in-excess endogenous metabolites. As SCLS and ABCs tubular transporters are involved in the tubular transfer of a vast number of small molecules, including inorganic ions, metabolites, nutrients and signaling molecules [9], they have a major role in controlling tissue and cell homeostasis (Figure 2). It is worth noting that the activity of the sodium-potassium pump (Na^+^/K^+^-ATPase), located at the basolateral pole, provides the electrochemical gradient necessary for tubular movements of electrolytes and solutes in all tubular segments [26]. As shown in Figure 2, tubular transporters coordinate the harmonized, bi-directional transport of their endogenous substrates. The vectorial reabsorption of glucose, as well as the cooperative interaction of some transporters of the amino acid transport system, illustrates this phenomenon. 

As well as their role in homeostasis described above, SLCs and ABCs transporters are also involved in detoxification processes by contributing to the renal handling of metabolic wastes, environmental chemicals and uremic toxins [27]. Uremic toxins are compounds normally eliminated by the kidney, which accumulate in the case of chronic kidney disease, causing several complications, including nephropathy [11]. The list of these molecules is available on the network of the European Uremic Toxin Workgroup (http://www.uremic-toxins.org). This list contains more than 150 substances derived from dietary protein breakdown, endogenous metabolic pathways or the gut microbiome [27]. They are divided into three classes based on their chemical properties: (i) small water-soluble, non-protein-bound solutes, with a molecular weight less than 500 Da (e.g., urea, creatinine, uric acid); (ii) middle size molecules (e.g., parathyroid hormone); and (iii) small protein-bound solutes (e.g., *p*-cresol, indoxyl sulfate, hippuric acid) [27,28]. Even if most of these toxins can be removed from the body by glomerular filtration, elimination of the protein-bound uremic toxins depends predominantly on tubular transporters’ activity [27]. Table 1 presents the main uremic toxins handled by multi-specific tubular transporters, of which OAT1 and OAT3 are the most important. Although numerous studies have documented the specificities of certain SLC transporters for these protein-bound uremic solutes/toxins, their apical efflux into urine and their excretion by ABC transporters are less well known.

Renal tubular transporters are also responsible for the handling of many drugs, some of which are used in kidney transplantation. Ivanyuk et al. recently reviewed current knowledge on the role and clinical relevance of tubular transporters in drug therapy [29]. Many other reviews are available for a comprehensive understanding of this topic, and interested readers are referred to corresponding references [9,10,18,30,31,32,33]. Table 1 presents a list of the main xenobiotics handled by renal transporters (Table 1).

## 4. Effects of IRI on Renal SLC and ABC Proximal Tubular Transporters

**Figure 3:**
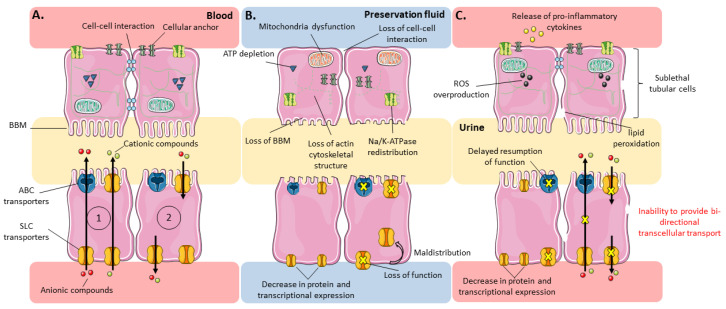
Evolution of subcellular structures and expression/function of tubular transporters under physiological and ischemia-reperfusion conditions focused on sublethal tubular cells. Evolution of subcellular structures (top) and expression/function of tubular transporters (bottom) under (**A**) physiological, (**B**) ischemic and (**C**) reperfusion conditions, focused on sublethal tubular cells. (1) Tubular secretion; (2) Tubular reabsorption. ABC and SLC transporters are distinguished by their shape and do not represent specific members of their respective families. Artwork was designed using Servier Medical Art by Servier licensed under CC BY 3.0 (https://smart.servier.com).

Proximal tubular cells are particularly sensitive to IR-induced injury [4,61,62,63] because of the high metabolic energy required for membrane transport of essential metabolites, their strong dependence on oxidative phosphorylation and their limited ability to utilize anaerobic glycolysis [61,62]. Numerous alterations affect tubular proximal cells during IRI [2,5,61,62], whether structural (e.g., actin cytoskeleton remodeling [64,65], brush-border membrane disruption [66], loss of cell polarity, loss of tight junctions [62] and rupture in the continuity of the phospholipid bilayer [61]) or functional (e.g., mitochondrial swelling and impaired mitochondrial respiratory capacity [62], deprivation in intracellular ATP [67], modification of calcium and sodium homeostasis [5]). It is thus relevant to hypothesize that cells with sub-lethal injuries have a reduced capacity for transcellular transport of their endogenous and exogenous substrates (Figure 3). Several studies actually provided insights in this matter by analyzing the effects on transporters expression and/or function of ischemia alone, or ischemia followed by reperfusion. Others have indirectly evaluated transport activity by measuring plasma or urinary metabolite content in conditions of, and after recovery from, I and/or IR.

### 4.1. Effects of Ischemia (I) and/or Ischemia-Reperfusion/Reoxygenation (IR) on Transporters’ Expression and/or Function

Several studies, conducted either in vitro or in vivo, have evaluated whether and how tubular transporters are modulated by I and/or IR. They mainly focused on the transport of electrolytes, metabolites and drugs and were all conducted at 37 °C, thus evaluating the effects of warm ischemia. In this section, we present the major tubular transporters whose activity or expression is modulated by IR. IRI and associated kidney dysfunction have numerous deleterious clinical consequences in transplant patients. However, allograft dysfunction is multifactorial and transporters only account for a small piece of the puzzle. Although it is very difficult to make direct links in such a complex process, there is evidence that transporters are involved in part of the electrolyte and acid-base disorders encountered after kidney transplantation [68]. Connections between transporter’s modulation and related consequences on graft outcomes or on toxins and xenobiotic accumulation are further discussed in the next chapters.

#### 4.1.1. Effects on Solute Carriers (SLC)

Several studies have analyzed the consequences of I or IR on Na^+^-dependent uptake of compounds by tubular cells. Brush-border membrane vesicles isolated from mouse kidneys subjected to different conditions of ischemia/reperfusion showed decreased ^32^Pi uptake after 15, 30 and 60 min of ischemia induced by renal arterial clamping. This correlated with a decrease in protein expression of the NaPi-II cotransporter. The uptake of ^32^Pi and NaPi-II expression only reappeared after 2 h of reperfusion following 30 min of ischemia, but was not restored when ischemia lasted 60 min [69]. 

In rats subjected to 15 and 30 min of renal ischemia, NHE3 mRNA in the kidney cortex significantly decreased after 12 h of reperfusion and was still low after 24 h. The transcriptional expression of NHE3 was less severely affected after 15 min clamping than 30 min^99^. Other authors showed decreased NHE3 transcripts and protein expression in rat cortex and medulla for up to 10 days after reperfusion, following 40 min of ischemia [70]. Kwon et al. examined the abundance of several Na^+^-coupled transporters in rats subjected to different ischemia (30, 40 and 60 min) and reperfusion (1–5 days) time durations. After 1 day of reperfusion following 30 min of ischemia, they saw a decrease in protein expression of NHE3, NaPi-IIa and Na^+^/K^+^-ATPase, paralleling an increase in urinary excretion of Na^+^. Five days after 30 min of ischemia, urinary excretion of Na^+^ normalized but the renal expression of Na^+^ transport proteins had incompletely recovered. Sixty minutes of ischemia induced stronger changes in these parameters on day 1, with no recovery on day 5 [71]. 

Several studies in rats have demonstrated that renal ischemia is also associated with a transient decrease in glucose transport activity on proximal tubule brush border vesicles, due to decreased SGLT2 expression at the apical membrane [72,73]. Glucose reabsorption was significantly reduced in the post-ischemia period, with partial recovery after 4 h [72]. 

Similar results were obtained with SLC transporters involved in xenobiotic clearance and detoxification processes [74]. Many studies showed decreased clearance of uremic toxins using a rat model of ischemic acute renal failure [75,76,77]. Matsuzaki et al. showed that IR (30-min bilateral clamping of renal arteries followed by 6, 12, 24 or 48 h of reperfusion) was associated with a significant increase in the concentration of indoxyl sulfate in serum, proportional to reperfusion time duration [77]. This paralleled a significant decrease in rOAT1 and rOAT3 mRNAs and protein expression. A decrease in the uptake of PAH and estrone sulfate was also observed in renal slices taken from these rats at 48 h of reperfusion, consistent with the decreased rOAT1 and rOAT3 protein expression at that time. Schneider et al. also showed that 45 min of renal ischemia is accompanied by a downregulation of protein and transcriptional expression of rOAT1 and rOAT3, with an early decrease (as early as 6 h after the start of reperfusion) followed by a partial recovery at 24 h and a complete restoration at 72 h [76]. These results were corroborated using an in vitro model of hypoxia-reoxygenation, showing that 2 h of hypoxia induced a significant decrease of basolateral fluorescein uptake 48 hours after reoxygenation [78].

Regarding organic cations, the renal clearance of tetraethylammonium (TEA) was significantly lower in rats exposed to IR (48 h of reperfusion following 30 min bilateral arterial clamping), as compared to control animals. In addition, the authors found a significant decrease in the uptake of TEA by renal slices isolated from these rats. However, the concentration of TEA in the kidney was significantly elevated in rats exposed to IR, suggesting a concomitant decrease of its efflux transport. This altered TEA transport was associated with significant decreases in rOCT2 and rMATE1 mRNAs and protein expression, the latter transporter being indeed the most severely affected [79]. A significant decrease in rOCT1 and rOCT2 mRNAs and protein expression was also observed 24 h after 45-min bilateral clamping of the renal arteries in rats [80]. The uptake of methyl-4-phenylpyridinium, a prototypical OCT substrate, was also reduced in the authors’ in vitro IRI model [80]. In mouse models of either syngenic or allogeneic kidney transplantation, a significant decrease in rOCT1 mRNA and protein expression was observed at day 4 post-transplantation in both conditions [81], whereas decreased rOCT2 mRNA and protein expression was only observed in allogeneic transplantation. This suggests a link between the immune response and the downregulation of some tubular transporters. 

In humans, Corrigan et al. determined the effect of post-ischemic lesions on PAH clearance and PAH extraction rate, by measuring renal blood flow using magnetic resonance imaging [82]. The study included 44 kidney transplant patients in whom PAH clearance was measured at 1–3 h and then at 7 days after transplantation. PAH clearance decreased independently of renal plasma flow, confirming that active proximal secretion of PAH is impaired in kidney transplant patients. This strongly suggests that organic anion transporters are dysfunctional after an ischemic insult, although their expression was not measured in this study. In this regard, Kwon et al. performed an immuno-histochemical analysis with confocal microscopy of hOAT1 on biopsies from 10 cadaveric donor renal allografts, taken 1 hour after reperfusion following a total ischemic time of 1574 ± 72 min [83]. They found diverse abnormal cell localizations of OAT1, characterized by variable patterns of misdistribution between basolateral membrane and cytoplasm. In a subset of patients in whom PAH net tubular secretion was measured at post-operative day 4 ± 1, a trend to more severe hOAT1 misdistribution was seen in patients with the lowest PAH clearances. Nonetheless, even subjects with misdistribution or absent hOAT1 were still able to secrete PAH, probably because hOAT3 is less severely altered by ischemia than hOAT1. 

#### 4.1.2. Effects on ABC Transporters

Contrary to SLCs, information on the effects of IR on renal ABC drug transporters is limited. In their above-described study showing decreased rOAT1,3 expression and function up to 48 h after reperfusion, Matsuzaki et al. found a transient increase in the amount of mRNA encoding Mdr1 after 6 and 12 h of reperfusion, with a return to basal values at 24 h. At 48 h of reperfusion, rMdr1 mRNA level and P-gp protein expression were identical to those of control animals [77]. Following 30 min bilateral clamping of renal arteries in a mouse model, Huls et al. found that some transporters genes were up-regulated and others downregulated 7 days after ischemia [84]. In particular, there was an increase in the mRNA levels of the *ABCB1*, *ABCB11* and *ABCC4* genes but a decrease for *abca3*, *abcc2* and *abcg2*. For each of them, the expression levels had returned to baseline at day 14. A significant decrease in the protein expression of *ABCA3*, *ABCB44* and *ABCB11* and *ABCC2* was observed 7 days after reperfusion, suggesting differential modulation of various ABC transporters during ischemia-reperfusion in the kidney. The opposite pattern between *ABCC4* and *ABCB11* transcripts and expression of the respective proteins suggests post-transcriptional regulatory mechanisms. We hypothesize that this differential regulation of ABC transporters during IR results from the regeneration process, which takes place after ischemic injury as an adaptive response to maintain homeostasis.

#### 4.1.3. Possible Mechanisms Underlying Membrane Transporter Dysfunction during I or I/R

As detailed above, experimental and clinical studies clearly show that IR induces decreased transport activities of most renal SLC transporters and has differential effects on ABC transporters. Reduced tubular uptake of substrates from blood along with up-regulation of P-gp and other ABC transporters has the potential to decrease the amount of potentially harmful substrates in the proximal tubular epithelium. However, after this brief quiescence, resumption of the activity of transporters in the post-ischemic phase ensures the entry of various metabolites essential to the viability of tubular cells. Overall, tubular transporters are mechanistically involved in cell dysfunction during IR but their restoration protects the organ after IR [85]. It is worth mentioning that the kinetics of transcriptional events and of protein expression generally differ. Most authors showed a rapid restoration of mRNAs encoding rOAT1 and rOAT3 and a slower recovery of protein expression after reperfusion [76]. In allograft recipients, kidney secretory function measured by PAH extraction is greatly reduced 1–3 h after reperfusion and generally restored within about 7 days, except in some patients who present with sustained acute kidney injury [82]. Several mechanisms might explain such time-dependent modulation of the expression and/or activity of tubular transporters under these conditions. 

Although most studies have linked decreased activity of SLCs to decreased expression at the transcriptional or protein level, we recalled above that SLC-mediated transport directly depends on the activity of the Na^+^/K^+^-ATPase pump, whose expression and activity is markedly depressed in the ischemic kidney [61,65,71]. The decreased inward Na^+^ gradient in renal tubular cells reduces the outward gradient of *α*-ketoglutarate and other di-tricarboxylates, and then the influx of OATs substrates. Regarding OCTs, the Na^+^/K^+^-ATPase defect reduces the inside-negative electric potential, which is the driving force of OC^+^ influx. The loss of function of NHE3 at the apical pole, which limits availability of the co-substrate necessary for MATEs-mediated transport, probably plays a role too. Similarly, ischemia-associated ATP depletion could be a cause of dysfunction of the ATP-dependent ABC transporters [25]. 

Transporter activity can also be regulated by modification of protein expression, posttranslational regulation, oligomerization, protein trafficking, epigenetics and non-genomic pathways, triggered by hormones and/or growth factors [14,86,87]. The mechanism governing the transcriptional regulation of transporters by IR is not fully understood but, as for other genes, it probably depends on specific transcription factors. The genomic response to hypoxia depends primarily on the Hypoxia Inducible Factor (HIF-1), which binds to Hypoxia-Response Elements (HRE) on the promoter of target genes and orchestrates their transcription [88]. Several studies demonstrated the importance of HIF in renal protection against IRI [89,90,91]. Other studies highlighted a potential link between HIF and transporters’ regulation [92,93]. Besides the HIF-1 pathway, the recently identified ischemia/reperfusion-inducible protein (IRIP) was shown to negatively regulate the activities of various transporters, including OCT1-2-3, MATE1, OAT1 and Pgp [94,95,96]. Some authors suggested that the abnormal expression and distribution of rat and human OAT1 may be related to the activation of protein kinase C (PKC), which downregulates hOAT1-mediated transport through carrier internalization from the cell membrane [83,97,98]. In line with this hypothesis, it was shown that prostaglandin-E2, a COX metabolite favored by IR, specifically downregulates OAT at the transcriptional level by acting via the E prostanoid receptor type 4 (EP4), subsequently activating PKA [99].

### 4.2. Effects of I and/or IR on Tubular Transport Functions as Evaluated by Metabolomics Studies

Several metabolomics analyses were conducted to study the consequences of I or IR in vivo. Based on the identification of metabolites differentially affected in the pre-ischemic, ischemic and reperfusion periods, this approach was used to elucidate the underlying biochemical processes and to identify biomarkers [100,101,102,103,104,105,106]. The metabolites usually found to be altered reflect the change in energetic pathways (i.e., a switch from the glycolytic pathway to fatty acid beta-oxidation feeding the TCA cycle), or belong to oxidative stress pathways [63]. However, as most of these metabolites are substrates of transporters, their concentration in graft preservation solutions, urine, plasma or renal tissue may also be related to tubular injuries and/or altered transporter function. 

Analysis of the preservation solution during hypothermic perfusion machine (HPM) or static cold storage (SCS) revealed that numerous metabolites, not initially present in the solutions, were detected less than 1 h after the begin of conservation period. There were also significant changes in the concentration of constituents of the preservation solutions. These changes may point out at products of metabolism being released from the kidney and substances being removed by the kidney to supply ongoing cell processes, respectively. This could be related to a rupture of the epithelium integrity, cell death or changes in subcellular energy production, but also to global and/or selective (dys)-function of tubular transport systems in ischemic conditions. By comparing, in paired porcine kidneys, the ratio of tissue-to-perfusion fluid levels of metabolites between SCS and HPM conditions, Nath et al. showed that, while a number of metabolites were released during storage in the two conditions, several (e.g., alanine, succinate, tyrosine and leucine) exhibited much less tissue accumulation with HPM than with SCS, suggesting that some cellular transport processes remained active during perfusion [107]. Similarly, rapid glutathione depletion in the conservation fluid during HPM is likely to reflect cellular uptake of this protective antioxidant. Another ex situ organ preservation method, called Normothermic Machine Perfusion (NMP), is the subject of renewed interest. It intends to mimic physiological conditions in the human body and has shown promising results [108,109]. However, to the best of our knowledge, no metabolomic study has yet been conducted on perfusion fluid coming from NMP. 

Recently, Stryjak et al. [106] studied the influence of long-term ischemia (2, 4, 6 and 21 h of static cold preservation) on tissue quality using an in situ kidney metabolomics analysis in rabbits. They found significant variation in the tissue content of numerous compounds as compared to the baseline. These metabolites belong to various biochemical pathways, including those involved in reactive oxygen species (ROS) production or amino-acid metabolism. This study confirms the time-dependent impact of ischemia on renal metabolic balance, as already observed for the expression and functionality of transporters. However, the lack of concomitant analysis of the perfusion fluid hinders drawing conclusions on the added contribution of altered transport versus pure metabolic disturbances. 

Wei et al. explored the metabolome in the plasma and in the kidney cortex and medulla from a mouse model of warm IR [101]. Various catabolites of amino acids and fatty acids (3-indoxyl sulfate, *p*-cresol sulfate, glycine- or acetyl-conjugates) normally excreted in urine, peaked in plasma as early as 2 h after reperfusion. This, together with the significant accumulation of urate in the kidney tissue, may reflect decreased tubular transport by OATs and MATEs, respectively. At the time of the most severe injury (48 h), markedly decreased concentrations of metabolites related to energetic (glucose, free fatty acids, amino acids), purine and other major metabolic pathways were found in the kidney tissue and plasma. This may primarily be due to the lack of oxygen and nutrient supply and to mitochondrial dysfunction, but one cannot rule out decreased tubular reabsorption of these metabolites. Rising levels of PGs in kidney tissue over reperfusion time [101] may reflect inflammation, a well-known component of IR, but also decreased tubular organic anion secretion. Finally, using unilateral clamping of renal artery in a swine model, Malagrino et al. found numerous metabolites in serum or urine able to discriminate between the pre-ischemic and ischemic periods [100]. Using pathway analysis, these metabolites were related to amino-acids degradation and to several molecular and cellular functions including, among others, lipid metabolism, biochemistry of small molecules, and molecular transport. By comparing nuclear protein content of ischemic and non-ischemic kidney tissue, the authors were able to rebuild a network of metabolic processes during IR. Unfortunately, they did not analyze whether or not membrane transporters were differentially modulated in ischemic vs. non-ischemic kidneys.

Altogether, these studies demonstrate that IR is accompanied by the disruption of several metabolic pathways (glucose, lipid and nucleotide metabolism, TCA cycle), which translates into variations of metabolomic profiles in biological fluids and/or tissues. Although the role that disruption of tubular transporters plays in some of the metabolomic variations observed has not been firmly demonstrated, it cannot be overlooked as they regulate cell exchanges and provide for kidney energy needs. 

## 5. Consequences of IR-Induced Modulation of Renal Transporters in the Post-Transplantation Period

The evolution of the expression and activity of renal tubular transporters SLCs and ABCs under the effect of IR in kidney transplantation is an important field of research. It may help to better understand and characterize tubular cell dysfunctions associated with IR and thus understand graft outcome. As presented above, SLCs and ABCs transporters contribute to the homeostasis of endogenous and/or exogenous substances and IR-induced modulation of their functions can (i) disrupt the reabsorption of essential nutrients (ii) cause cytotoxicity in proximal tubular cells (iii) promote local inflammation during reperfusion (iv) induce disturbances in other organs of the body, or (v) modify drug pharmacokinetics and/or toxicity, all these processes being potentially deleterious for the graft and the recipient.

### 5.1. Alteration of the Graft Itself

As described previously, IR is accompanied by an apical redistribution of the Na^+^/K^+^-ATPase pump and of NHE3, leading to decreased apical reabsorption of Na^+^ [110]. Higher urinary concentration of Na^+^ results in decreased glomerular filtration through the tubulo-glomerular feedback that helps to limit Na^+^ and fluid loss. This phenomenon may serve to protect the vulnerable tubules, but reduction of glomerular filtration rate favors renal failure [63]. Tubular reabsorption of Na^+^ is normally followed by osmotic reabsorption of water by aquaporins 1 and 3, which have also been found to be dysregulated by IR [111]. In addition, IR disrupts the close coordination between Na^+^ absorption at the apical membrane and its efflux at the basolateral membrane, which is needed to prevent increased osmotic pressure and swelling of the cell. 

In a more general context, acute loss of renal function in AKI results in multiple disturbances in fluid, electrolyte and acid-base homeostasis. For example, the kidneys normally excrete fixed non-volatile acids resulting from the catabolism of dietary proteins to maintain acid–base homeostasis. AKI is often associated with metabolic acidosis showing a disruption of this balance, linked to the retention of organic anions [112].

During tissue reoxygenation, the ROS overproduction is deleterious to proximal tubular cells and the graft [8]. Radical scavenging is normally accomplished by intracellular antioxidants, including many TCA cycle metabolites that indirectly help to maintain sufficient glutathione levels by increasing NADPH production [113]. Ergothionein has antioxidant properties that contribute in decreasing ROS production and local inflammation in the post-transplant period [114]. L-carnitine also has antioxidant and protective effects in tissues with oxidative damage and its administration 30 min before reperfusion had protective effects against I/R-induced AKI [115,116,117]. A disruption of the functionality of the OCTNs, OATs and NaDCs transporters during the post-transplant period can disrupt reabsorption of these antioxidants from urine. A study showed that succinate is responsible for the production of mitochondrial ROS during reperfusion [118]. Hypoxia-dependent succinate accumulation is also associated with a shift in the equilibrium of many TCA cycle metabolites, including oxaloacetate, fumarate, α-ketoglutarate, aspartate, citrate and isocitrate [119]. The equilibrium between elimination and reabsorption of TCA cycle intermediates in ischemic conditions thus appears to be an important phenomenon in reperfusion lesions. OAT1, OAT2, OAT3 and NaDC3 at the basolateral pole and NaDC1 at the apical membrane ensure intra/extra-cellular exchanges of these intermediaries at the tubular level. A recent study in mice treated with a continuous infusion of β-hydroxybutyrate (a ketone body, substrate of OAT1) before and after IRI, showed that it has a protective effect against acute renal IR injuries [120]. It implies that, without supplementation, decreased intracellular availability of β-hydroxybutyrate may favor tubular injury. Interestingly, the renoprotective effect sustained for up to 24 h after infusion is stopped. The authors postulated that it resulted from long-acting effects of β-hydroxybutyrate on epigenetic regulation of several genes controlling pyroptosis. We may also hypothesize a role for tubular transporters, acting in cooperation to maintain high intracellular levels of β-hydroxybutyrate. Overall, we hypothesize that perturbation of transmembrane transport, responsible for the intracellular disequilibrium of metabolites necessary for the energetic pathways and/or the protection against free radicals, is a key mechanism of IRI-induced AKI. 

Other authors highlighted the importance of epithelial cell maladaptive repair mechanisms in the promotion of pathological phenotypes in kidney transplant recipients, characterized among others by interstitial fibrosis, tubular atrophy and capillary rarefaction [121,122,123]. While tubular epithelium repair may obviously improve tubular transport functions, tubular transporters might also play a role in tubular epithelium repair [14,124] by maintaining homeostasis in sublethal tubular cells, thus favoring progressive tubular repopulation with nonlethally injured cells [125,126]. 

Besides, recent studies suggested that transporters of the SLC22 family participate in a wide network of communication between various systems of the organism. Based on the distribution of OATs across organs and on the diversity and specificities of their substrates, a phenomenon of “remote sensing and signaling” was suggested, by which cooperation between transporters with different tissue expression patterns helps to maintain whole-body homeostasis [9,127,128,129]. The presence of a multi-scale network of transporters, together with possible compensatory up-regulation between transporters at the renal level [130], could have repercussions in normal physiology and pathophysiology of the transplanted organ and of the whole organism [61]. In this sense, a frequent consequence of reperfusion after localized tissue ischemia is injury to other organ systems, so-called distant or remote organ injury (ROI) [131,132,133], including hepatic changes after renal IR injury [134,135].

### 5.2. Accumulation of Toxic Substances

IR-induced modulation of tubular renal transporters leads to an accumulation of toxic substances in the post-transplantation period, which may have prejudicial consequences on graft function or outcome. Understanding how these transporters are modulated along time and deciphering the consequences of increased exposure to uremic toxins or other toxic compounds may be useful for the monitoring of renal transplant recipients and the development of new therapeutic strategies. As previously mentioned in this paper, many studies showed a decreased clearance of uremic toxins, either anionic (e.g., indoxyl sulfate, PAH, estrone sulfate) or cationic (TEA, guanidine), that paralleled the decreased expression of their corresponding transporters [76,77,79,100]. In turn, their clearance also normalized in parallel to the transporters activity’s restoration [76,77].

In recent years, important progress has been made in the understanding and characterization of the toxicity of uremic toxins, especially for *p*-cresol and indoxyl sulfate. An in vitro study evaluated the effects on endothelial cell proliferation in a large panel of uremic solutes (guanidine compounds, polyamines, oxalate, myoinositol, urea, uric acid, creatinine, indoxyl sulfate, indole-3-acetic acid, *p*-cresol, hippuric acid, and homocysteine). It demonstrated that *p*-cresol and indoxyl sulfate reduced endothelial cell proliferation and wound repair, in a dose-dependent manner for *p*-cresol [136]. Indoxyl sulfate, at concentrations found in uremic patients, also induces the production of oxidative stress radicals in endothelial cells [137]. As recently described by Huang et al., PAH also promotes endothelial dysfunction by increasing ROS production, thus possibly accelerating tubular damage [138]. Moreover, uremic toxins can also have a direct impact on the expression of tubular cell transporters. Indeed, Brandoni et al. found that exposure of a suspension of proximal tubular cells to urea led to a significant decrease in OAT1 and OAT3 protein abundance in cell membranes, in a concentration-dependent manner [139]. Therefore, the direct effect of uremic toxins on transporters function, whose expression is already decreased, may have a synergistic effect on their accumulation, together with that of other endogenous or exogenous substrates. 

In renal transplant patients, evaluating the particular role of altered tubular transport on graft dysfunction and rejection is difficult, as it is far from being the sole mechanism involved in these complications. Nevertheless, Knoflach et al. showed that PAH concentrations increased in patients who experienced acute renal allograft rejection and decreased rapidly after successful antirejection treatment [140]. Similarly, Corrigan et al. confirmed that secretion of PAH is impaired for at least 7 days, even after the onset of GFR recovery [82]. Impaired secretion of PAH may arise from decreased transporter expression but may also involve a competitive inhibitory mechanism. Indeed, uremic toxins and other products normally eliminated by the kidneys (or by hemodialysis) accumulate in blood in the first few hours or days after transplantation. These toxins can limit the renal excretion of each other by competitively inhibiting transporters.

In a rat model of chronic renal failure (CRF), the overload of PAH and indoleacetic acid accelerated the loss of kidney function, glomerular sclerosis and tubulointerstitial injury [141]. Numerous studies in patients with CRF showed that uremic toxin accumulation leads to various complications including endothelial senescence [142], atherogenesis [143], cardiovascular diseases [144,145] or increased risk of all-cause mortality [144,146]. In CRF, these toxins accumulate for very long periods, making their toxicity particularly critical. As their accumulation in the transplanted patient is reversible, owing to the restoration of glomerular filtration and transporter’s function, their deleterious effects should be less important. However, these putative uremic toxins are poorly filtered across dialysis membranes because they are protein bound and current dialysis therapy does not correct the full spectrum of uremic toxicity [27]. Therefore, in the context of IR-induced AKI, residual tubular transporter function may contribute to the attenuation of acute kidney dysfunction in the immediate post-transplant period.

### 5.3. Impact on Drugs Used in Transplanted Patients

Downregulation of renal tubular transporters induced by IR, together with subsequent accumulation of uremic toxins may modulate the pharmacokinetics of several drugs, leading to decreased clearance and risk of increased toxicity in the early post-transplantation period. Functional alteration in renal excretion is of particular clinical importance for drugs with a narrow therapeutic range, such as immunosuppressants. 

Generally, inhibition of basolateral uptake transporters causes an increase in the systemic concentration of their substrates, while inhibition of apical efflux transporters can cause an increase in intracellular concentrations, leading to possible nephrotoxicity. However, information about pharmacokinetic alterations resulting from decreased function of renal tubular transporters for drugs used in renal transplantation is limited. Tacrolimus, cyclosporine, sirolimus and everolimus undergo hepatic metabolism and are mainly eliminated in the bile. Others, like corticosteroids or mycophenolate mofetil, are conjugated in the liver and subsequently eliminated by the kidney, as inactive metabolites. This suggests that alteration of renal transporters should not be a major factor involved in the pharmacokinetics or toxicity of the parent compounds. However, renal tubular transporters are partially involved in the urine excretion of the unchanged fraction of tacrolimus and cyclosporin that are substrates of P-gp [10,29,32], of corticosteroids conjugates that are substrates of P-gp, BCRP and MATEs [10,29,32,147], and of mycophenolic acid glucuronide, a substrate of OAT3 [52] and MRP2 [148]. Moreover, as stated above, the hepatic disposition of these drugs can be impaired in renal transplantation due to competitive mechanisms arising from uremic toxin accumulation. Thus, the potential effect of renal tubular transporter’s alteration on these drugs, albeit indirect for a large part, should not be ignored.

Some studies evaluated the particular role of renal P-gp on calcineurin inhibitors’ toxicity in kidney transplantation, with controversial results [149,150,151,152]. In a prospective cohort of 252 adult renal allograft recipients receiving tacrolimus, Naesens et al. found that the absence of P-gp expression (evaluated by immunohistochemistry) and the combined donor-recipient homozygosity for the c.C3435T variant in *ABCB1* gene (possibly responsible for altered conformation and function of P-gp [153]) were associated with more chronic allograft damage [149]. The presence of this variant in the donor has also been associated with cyclosporine nephrotoxicity in a case-control study [150]. However, other authors found no such association [152] or even the opposite [151]. Due to its wide expression on numerous tissues, it is very difficult to evaluate the part of tubular P-gp in the global role it may have on calcineurin inhibitors’ pharmacokinetics. Hence, to date, no clear conclusion can be drawn about IR-induced P-gp dysfunction and the risk of calcineurin inhibitors’ toxicity.

Interestingly, immunosuppressants also have inhibitory effects on various transporters. OAT1 and OAT3 are inhibited by mycophenolic acid, while MRP2 and MRP4 are inhibited by mycophenolic acid, tacrolimus, cyclosporine and hydrocortisone [154,155], suggesting that the fraction of these compounds reaching the kidney could have a synergistic effect with IR on the decreased function of tubular transporters. As mentioned before, a reduced expression of tubular OCT1 and OCT2 was observed after allogeneic kidney transplantation in rats [81]. The authors confirmed that cyclosporine A, without being itself a substrate of OCTs, had inhibitory effects on their activity and could also possibly lead to drug–drug interactions. 

It is well known that sirolimus and calcineurin inhibitors, mostly cyclosporine, are involved in numerous drug–drug interactions [156,157,158,159]. An in vitro study conducted on human renal epithelial cells showed that P-gp protects renal epithelial cells from cyclosporine toxicity and that sirolimus inhibits P-gp-mediated efflux of cyclosporine, leading to intracellular accumulation of the drug. This suggests that the renal P-gp could play a role in the potentiation of cyclosporine nephrotoxicity by sirolimus [160]. 

It has been demonstrated that cyclosporine increases mycophenolic acid glucuronide exposure via the inhibition of its biliary excretion by MRP2 [161]. However, El-Sheik et al. demonstrated on rat kidney homogenates that cyclosporine also interferes with mycophenolic acid renal excretion by inhibiting its renal glucuronidation and the subsequent MRP2-mediated transport of mycophenolic acid glucuronide [162].

Thus, kidney transplant recipients are exposed to transporter-mediated drug–drug interactions but also drug-metabolites and drug-toxins interactions, which can amplify the consequences of IR-induced transporter dysfunction [163].

Pharmacokinetic consequences of impaired transporter expression have been studied, or can be anticipated, for drugs other than immunosuppressors. For example, the AUC and the half-life of famotidine were two-fold higher in IR rats than in sham operated controls, in line with downregulation of OCT2 and MATE1 [79]. Regarding anionic drugs, Sakurai et al. showed that cefazolin elimination correlated with *OAT3* mRNA levels in patients with renal disease [164]. Concerning the antibiotics commonly used in renal transplantation, trimethoprim, generally combined with sulfamethoxazole, is eliminated unchanged by renal excretion and is a substrate of MATE1 and MATE2 [165], suggesting that intrarenal accumulation of this drug could occur in response to their downregulation. On the contrary, downregulation of OATs at the basolateral membrane could decrease the renal uptake and accumulation of antivirals such as acyclovir, thereby reducing tubular toxicity. Generally speaking, IR-induced transporter’ alterations may impair the pharmacokinetics of numerous drugs, independently of, or in addition to, altered glomerular filtration.

## 6. Modulation of Transporter’s Function for Improving Graft Outcome

Different therapeutic approaches were proposed to reduce renal IRI, targeting various stages of transplantation: (i) before transplantation, by pre-treatment of livings donor (mostly dietary preconditioning) or conditioning of deceased donors before organ harvesting; (ii) during graft preservation, by using hypothermic or normothermic perfusion machines (the latter necessitating the use of an oxygen carrier) or by adding therapeutic agents to the preservation fluid; (iii) after transplantation, by modulating the recipient’ treatment [166]. 

Storage on NMP, which maintains organs at physiological temperature, restores normal cellular processes and allows kidneys to maintain their function. Hosgood and Nicholson found that 1 h of NMP improved early graft function of kidneys from extended criteria donors [167]. Moreover, Kaths et al. showed in a porcine model that prolonged periods of NMP improved graft function with significantly lower serum creatinine values and significantly higher creatinine clearance when compared with SCS [168]. This conservation technology enhances the functionality of the graft in the post-transplant period and could better preserve tubular transporters’ activity prior to reperfusion. However, as NPM is still technologically challenging with only limited pilot human studies available up until now [169], its effects on membrane transporters has not yet been evaluated.

While many therapeutic agents have been tested and are effective in animals to prevent or combat IRI, none has been implanted in routine clinical practice yet [166,170]. Most of them target major pathways of IR such as inflammation, energetic metabolism, oxidative stress or coagulation and rely on supplementation of the preservation fluid with a therapeutic agent, gene therapy or cell therapies [171]. Other studies presented below and summarized in Table 2 evaluated how the harmful effects of IR can be minimized and renal recovery improved through modulation of transporters function (Table 2) [172,173,174,175,176,177,178,179,180,181]. Therefore, future research should be directed to increase the contribution of IR-induced transporter’s modulation in order to improve the implementation of new therapeutics or the development of new diagnostic markers.

Gong et al. demonstrated in vitro that truncated Na^+^/K^+^-ATPase β (tNKAβ) could activate the NKA α subunit and thus enhance the activity of the Na^+^/K^+^-ATPase. They then showed on a rat model that the tNKAβ-treated group (2 h before reperfusion) exhibited significant improvement in renal function, with lower serum creatinine and blood urea nitrogen (BUN) levels on postoperative days 1–6 [172]. Belliard et al. showed in rat heart preparations exposed to 30 min of ischemia followed by 30 min of reperfusion that preconditioning with a sub-inotropic concentration of the cardiac glycoside ouabain prevented ischemia-induced alterations of the Na^+^/K^+^-ATPase and was associated with better contractile function [173]. Schneider et al. also demonstrated that, after bilateral clamping of renal arteries in rats, intraperitoneal injection of indomethacin (inhibiting the IR-dependent increase in PGE_2_) prevented the downregulation of rOAT1 and rOAT3 transporter expression during reperfusion and resulted in an increased PAH net excretion [174]. They also demonstrated that the beneficial effects of indomethacin on renal function in IR conditions were related to the increased expression of the OAT1 and OAT3 transporters [85]. Similarly, intravenous administrations of meclofenamate in a rat model of IR had a nephroprotective effect by restoring the renal expression of rOAT1 and rOAT3 [175]. However, Yamashita et al. demonstrated in uninephrectomized mice undergoing left renal artery clamping, that pre-ischemic treatment (but not post-ischemic) with 5-(*N*-ethyl-*N*-isopropyl) amiloride, an inhibitor of NHE, attenuated IR-induced renal dysfunction [177]. NHE3 seemed to be involved in the development and progression of post-ischemic renal injury, probably by increasing Na^+^/Ca^2+^ intracellular concentrations during ischemia. Similar results were found with a NHE3 selective inhibitor (S3226) in a rat model of IR [178]. 

Genetic deletion of SGLT1 was recently found to improve recovery of kidney function on day 14 in a mouse model of renal IR and was associated with a reduced tubular injury score [182]. These studies clearly demonstrate that modulation of transporter activity during ischemic periods and/or reperfusion is accompanied by an attenuation of ischemic damage. However, the nature of this beneficial modification, whether accentuation or inhibition of activity, may depend on the transporters and more precisely on substrate specificity for the transporters.

Other potential therapies could depend on tubular transporter function. Side population (SP) cells, a stem-cell rich population found in the kidney that constitutively expresses BCRP, significantly improved renal function after bilateral clamping of renal arteries in mice [179]. The therapeutic potential of SP cells was reduced by pretreatment with verapamil, an inhibitor of BCRP, suggesting that this transporter contributes to the nephroprotective effect of SP. Kieran et al. evaluated the modification of the transcriptomic response to renal IRI by a lipoxin analog previously found to have nephroprotective effects in a murine model of IRI [180]. They found that this analog could restore the expression of many genes either downregulated or upregulated by IR. Although the effect of this lipoxin analog is not limited to a specific family of genes, this study showed that the expression level of renal transporters such as OCT1 or aquaporin 1 was restored. The PKC inhibitor, sotrastaurin, ameliorated ischemia-reperfusion organ damage and promoted cytoprotection in a model of extended renal cold preservation followed by transplantation [183]. It is interesting to remember that activation of PKC downregulates hOAT1-mediated transport through transporter internalization from the cell membrane [83,97,98], and to put forward the hypothesis that the beneficial effects observed in this study could be related to a restoration or non-loss of function of OAT1. 

Some years ago, pravastatin was shown to reduce the levels of specific uremic toxins (asymmetric dimethylarginine, guanidine succinate and trans-aconitate) in the plasma [181]. After administration of pravastatin, the mRNA level of rat slco4c1 was significantly increased in the kidney. Similarly, the application of pravastatin potentiated (by 1.73-fold) the *SLCO4C1* (OATP4C1) mRNA in human kidney proximal cells. Altogether, these data suggest that pravastatin increased ADMA and *trans*-aconitate excretion in the proximal tubules, via SLCO4C1 up-regulation. The authors showed that statins function as a nuclear receptor ligand, recruiting the AhR-XRE system and upregulating SLCO4C1 transcription. Whether this mechanism also applies to other kidney transporters has not yet been studied further. Another transcriptomics therapeutic target of interest is HIF-1. Pharmacological induction of HIF can be obtained using molecules that stabilize HIF or that inactivate prolyl hydroxylase domains (PHD) involved in HIF-1 degradation in the proteasome [86,184,185,186]. Interestingly, Hagos et al. found that PHD inhibitors are taken up into the tubular cells by OAT1 and OAT3 and released in urine by OAT4 [86]. This suggests that the downregulation of OATs during I/R could modulate the effectiveness of therapeutic agents like PHD inhibitors. Hence, therapeutic strategies aiming to modulate transcriptional or posttranscriptional mechanisms in tubular cells using pharmacological agents are potentially limited by associated factors [63]. 

## 7. Conclusions

IRI is a complex and important problem in kidney transplantation. Development of therapeutic strategies is critical due to the increasing use of marginal grafts, more susceptible to the injury processes. Elucidating the pathophysiological mechanisms of IRI may allow designing potential therapies. In this paper, we have assessed the impact of IR on major SLC and ABC transporters by selecting the most relevant papers published on this topic. Most of them showed that SLCs are downregulated during ischemic periods and recover with a delay upon reperfusion. In contrast, the literature points to a lack of knowledge regarding ABC transporters. Although we did not conduct a true systematic review to find all the relevant studies on the topic, we made a thorough analysis of the literature and completed the search by reading the most relevant references cited in these papers. This work allowed us to gather the still fragmentary knowledge concerning the impact of the IR on the tubular transporters. However, improved understanding of the modulation of these transporters by IR would contribute to better characterizing its clinical impact and anticipating renal function recovery. In our opinion, altered functionality of renal tubule transporters by IR and subsequent disruptions in the equilibrium of endogenous and exogenous substrates may participate in lesions that occur during kidney transplantation procedure. Protection and/or restoration of transport activity could be part of multiple treatment strategy at various time points during the donation, preservation and transplantation to reduce IRI and its consequences. It may represent an important research area for the improvement of short and long-term graft outcomes.

## Figures and Tables

**Table 1:** Main endogenous metabolites, uremic toxins and drugs handled by renal tubular transporters.

Part 1—Transporters of Endogenous Metabolites					
Transporter	Gene	Location	Transport Mode	Endogenous Substrates	References
*Inorganic Ions*					
NaPi-IIa	*SLC34A1*	Apical	Na^+^/phosphate co-transporter	Na^+^; Pi	[24]
NaPi-IIc	*SLC34A3*	Apical	Na^+^/phosphate co-transporter	Na^+^; Pi	
NHE3	*SLC9A3*	Apical	Na^+^/H^+^ exchanger	Na^+^; H^+^	[34]
NBCe1	*SLC4A4*	Basolateral	Na^+^/HCO_3_^−^ co-transporter	Na^+^; HCO_3_^−^	[35]
KCC3	*SLC12A6*	Basolateral	K^+^-Cl^−^ cotransporter	K^+^; Cl^−^	[36]
KCC4	*SLC12A7*	Basolateral	K^+^-Cl^−^ cotransporter	K^+^; Cl^−^	
NaS1	*SLC13A1*	Apical	Na^+^-SO_4_^2−^-cotransporter	Na^+^; SO_4_^2−^	[37]
Sat1	*SLC26A1*	Basolateral	sulfate anion transporter	SO_4_^2−^	
*Glucose*					
SGLT1	*SLC5A1*	Apical	Na^+^/glucose co-transporter	Na^+^; glucose	[38]
SGLT2	*SLC5A2*	Apical	Na^+^/glucose co-transporter	Na^+^; glucose	
GLUT1	*SLC2A1*	Basolateral	passive transport	glucose	
GLUT2	*SLC2A2*	Basolateral	passive transport	glucose	
*Peptides, amino acids*					
PEPT1	*SLC15A1*	Apical	H^+^/oligopeptide cotransporters	oligopeptides	[39]
PEPT2	*SLC15A2*	Apical	H^+^/oligopeptide cotransporters	oligopeptides	
*Neutral amino acids (AA0)*					
B^0^AT1	*SLC6A19*	Apical	Na^+^-neutral amino acid co-transporter	AA^0^	[40]
TauT	*SLC6A6*	Apical	Na^+^-dependent co-transporter	taurine; β-alanine; (GABA)	[40,41]
IMINO^B^	*SLC6A20*	Apical	Na^+^-Cl^−^-dependent co-transporter	proline; hydroxyproline	[40]
PAT2	*SLC36A2*	Apical	H^+^-dependent symporter	glycine; alanine; proline	[40]
LAT2-4F2hc	*SLC7A8/SLC3A2*	Basolateral	antiporter	ARO; AA^0^	[42,43]
TAT1	*SLC6A10*	Basolateral	uniporter	ARO	
*Cationic amino acids (AA+)*					
rBAT/b^0,+^AT	*SLC3A1/SLC7A9*	Apical	amino acids exchanger	AA^+^; AA^0^	[44,45]
4F2hc/y^+^LAT1	*SLC3A2/SLC7A7*	Basolateral	Na^+^-dependent amino acids exchanger	AA^+^; AA^0^	[46]
*Anionic amino acids (AA−)*					
EAAT3	*SLC1A1*	Basolateral	K^+^-Na^+^/H^+^/amino acids exhanger	l-glutamate; l-aspartate	[40]
*Tricarboxylic acid (TCA) intermediates*					
NaDC1	*SLC13A2*	Apical	Na^+^/di-tricarboxylate co-transporter	TCA cycle (e.g., succinate, citrate, α-ketoglutarate, fumarate)	[47]
NaDC3	*SLC13A3*	Basolateral	Na^+^/di-tricarboxylate co-transporter	TCA cycle (e.g., succinate, citrate, α-ketoglutarate, fumarate)	
SMCT1	*SLC5A8*	Apical	high-affinity Na^+^-coupled co-transporter	lactate; pyruvate; nicotinate	[48]
SMCT2	*SLC5A12*	Apical	low-affinity Na^+^-coupled co-transporter	lactate; pyruvate; nicotinate	
**Part 2–Transporters of Uremic Toxins and Drugs**					
**Transporter**	**Endogenous Substrates**		**Uremic Toxins**		**Drugs**
*Organic anions (OA^−^)*					
OAT Family					
OAT1; OAT2; OAT3; OAT4	mono-carboxylates (e.g., butyrate, lactate, propionate, pyruvate, 3-hydroxybutyrate, benzoate, 3-hydroxypropionate, 3-hydroxyisobutyrate); di-carboxylates (e.g., α-ketoglutarate, *N*-acetylaspartate); short-chain fatty acids (e.g., hexanoate, heptanoate and octanoate); prostaglandins (PGE2 and PGF2α); cyclic nucleotides (cAMP and cGMP); folate; nicotinate; tryptophan metabolites (quinolinate and kynurenate); purine metabolites (xanthine and hypoxanthine); adenine, adenosine, cytidine, guanidine, guanosine, inosine; hormones (estrone-3-sulfate, 17β-estradiol-3-sulfate, ß-estradiol-3-sulfate, β-estradiol-3,7-disulfate and DHEAS) [22,49,50]		uremic toxins (indoxyl sulfate, *p*-cresol, *p*-cresyl sulfate, indole-acetate, uric acid, creatinine, kynurenic acid, orotic acid, benzoate, trimethylamine *N*-Oxide (TMAO), 3-carboxy-4-methyl-5-propyl-2-furanpropionate (CMPF)); environmental toxins (mercury conjugate, ochratoxin A and aristolochic acid) [9,27,28,51]		Antivirals (adefovir, tenofovir, aciclovir, ciclofovir, cidofovir, lamivudine, stavudine); Non steroidals anti-inflammatory; Methotrexate; Diuretics (furosemide, bumetanide, hydrochlorothiazide); Angiotensin II receptors blockers (candesartan, valsartan, losartan…); β-lactams (penicillins chepalosporines…); Other antibiotics (tetracycline, ciprofloxacine); H2-antihistaminics (cimetidine, ranitidine, famotidine…); ACE inhibitors (captopril, quinaprine); HMG-coA reductor inhibitors (fluvastatin, pravastatin, simvastatin); Oral antidiabetics (glibenclamide…); mycophenolic acid glucuronide; Uricosuric-drugs (probenecid, benzbromarone) [9,29,30,52]
OATP Family					
OATP4C1	thyroid hormones (T^3^), cAMP [50]		asymmetric dimethylarginine (ADMA), guanidine succinate (GSA), and trans-aconitate [27]		Digoxin, ouabaine, methotrexate [29]
MRP Family					
MRP2; MRP4	glutathione, conjugated glutathione; bilirubin glucuronides and other metabolites (LTC4, E217βG, reduced glutathione (GSH)); cyclic nucleotides and ADP; prostaglandins (PGE1, PGE2); thromboxane B2 (TBX2); proinflammatory leukotrienes B4 et C4 (LTB4, LTC4); estradiol glucuronide (E217βG); folic acid [53,54]		kynurenic acid, probably indoxyl sulfate and hippuric acid [27]		Antivirals (tenofovir); Non steroidals anti-inflammatory; Methotrexate; Diuretics (furosemide); Angiotensin II receptors blockers; β-lactams [29]
BCRP Family					
BCRP	Vitamins (folic acid, B2, K3) estrone-3 sulfate, dehydroepiandrosterone sulfate, E217βG [55]		Urate, kynurenic acid, indoxyl sulfate, hippuric acid, *p*-cresyl sulfate and *p*-cresyl glucuronide [27,56,57]		Corticosteroids conjugates; Antineoplasics (mitoxantrone, methotrexate, irinotecan…); Antiretroviral nucleosides (AZT, lamivudine…); Fluoroquinolones; Statins (rosuvastatin); sulfasalazine; ITK (imatinib, gefitinib, nilotinib) [29,55]
*Organic cations (OC^+^)*					
OCT Family					
OCT2	choline, thiamine, l-carnitine [31]		polyamine uremic toxins (e.g., cadaverine, putrescine, spermine and spermidine); guanidino group (e.g., creatinine, guanidine or methylguanidine) [27,58]		Metformin; Cisplatin; H2-antihistaminics (cimetidine, famotidine, ranitidine…); antivirals; β blockers (pindolol…); Calcium channel blockers; Antiarrhythmics (procainamide, dofetilide…); Antimalarials; Varenicline; Amantadine; Memantine [29,31]
OCTN1; OCTN2	ergothionein; l-carnitine, choline [22,59]				Verapamil; Quinidine; Gabapentine; β-lactams (cephaloridine, cefepime); Valproic acid [10,29]
MATE Family					
MATE1; MATE2	6β-hydroxycortisol, E3S, *N*-methylnicotinamide (NMN), l-Arginine and thiamine [60]		guanidine, creatinine and ADMA [60]		Corticosteroids; Metformin; Cimetidine; Platinum compounds; Antibiotics (cefalexine, cephradine); H2-antihistaminics (cimetidine); Memantine [10,29]
MDR Family					
MDR1/P-gp	Anticancer drugs (methotrexate, anthacyclines, campthecins, taxanes…); Digoxin; Cardiovascular agents (valsartan, quinidine, nifedipine, verapamil); Immunosuppressant (ciclosporine, tacrolimus); Corticosteroids (corticosterone, dexamethasone); Antibactrial (erythromycine); Antiretrovirals (ritonavir, saquinavir); Phenobarbital; Phenytoin; Statins (lovastatin, simvastatin); H1-antihistaminic (fexofenadine); Anticoagulants (dabigatran); ITK inhibitors (imatinib, gefitinib); Antiepileptics (Topiramate); Analgesics (morphine) [9,10,29,32]				

Non-exhaustive list of endogenous and exogenous substrates handled by tubular transporters. Not all substrates of the OAT family are transported by each member of this family. For P-gp, we have represented only the xenobiotics transported.

**Table 2:** Summary of treatments used against Ischemia-Reperfusion-Injury, acting through modulation of transporters activities.

Treatment	Action	Model (Species/Organ)	References
tNKA(β)	Enhances Na^+^/K^+^-ATPase activity	Rat/Kidney	[172]
Ouabain	Preserves Na^+^/K^+^-ATPase activity	Rat/Heart	[173]
Indomethacin	Prevents OAT1 and OAT3 downregulation, increases PAH excretion	Rat/Kidney	[174]
Meclofenamate	Prevents OAT1 and OAT3 downregulation (induced by Protaglandin E2), Decreases indoxyl-sulfate hepatic production	Rat/Kidney, Liver	[175]
Ceftriaxone	Upregulates and increases SGLT1 activity	Rat/Brain	[176]
5-(*N*-ethyl-*N*-isopropyl) amiloride	Inhibits NHE	Mice/Kidney	[177]
S3226	Inhibits NHE3	Rat/Kidney	[178]
Side population cells	Cells which constitutively express BCRP	Mice/Kidney	[179]
Liopoxin analog	Restores expression of genes differentially expressed by IRI, including OCT1 and aquaporin 1	Mice/Kidney	[180]
Pravastatin	Upregulates OATP4C1	Rat/Kidney	[181]

## References

[1] Wong G., Teixeira-Pinto A., Chapman J.R., Craig J.C., Pleass H., McDonald S., Lim W.H. (2017). The Impact of Total Ischemic Time, Donor Age and the Pathway of Donor Death on Graft Outcomes After Deceased Donor Kidney Transplantation. Transplantation.

[2] Chen C.-C., Chapman W.C., Hanto D.W. (2015). Ischemia-reperfusion injury in kidney transplantation. Front. Biosci. (Elite Ed.).

[3] Menke J., Sollinger D., Schamberger B., Heemann U., Lutz J. (2014). The effect of ischemia/reperfusion on the kidney graft. Curr. Opin. Organ Transplant..

[4] Ponticelli C. (2014). Ischaemia-reperfusion injury: A major protagonist in kidney transplantation. Nephrol. Dial. Transplant..

[5] Salvadori M., Rosso G., Bertoni E. (2015). Update on ischemia-reperfusion injury in kidney transplantation: Pathogenesis and treatment. World J. Transplant..

[6] Venkatachalam M.A., Weinberg J.M., Kriz W., Bidani A.K. (2015). Failed Tubule Recovery, AKI-CKD Transition, and Kidney Disease Progression. J. Am. Soc. Nephrol..

[7] Zhao H., Alam A., Soo A.P., George A.J.T., Ma D. (2018). Ischemia-Reperfusion Injury Reduces Long Term Renal Graft Survival: Mechanism and Beyond. EBioMedicine.

[8] Nieuwenhuijs-Moeke G.J., Pischke S.E., Berger S.P., Sanders J.S.F., Pol R.A., Struys M.M.R.F., Ploeg R.J., Leuvenink H.G.D. (2020). Ischemia and Reperfusion Injury in Kidney Transplantation: Relevant Mechanisms in Injury and Repair. J. Clin. Med..

[9] Nigam S.K., Wu W., Bush K.T., Hoenig M.P., Blantz R.C., Bhatnagar V. (2015). Handling of Drugs, Metabolites, and Uremic Toxins by Kidney Proximal Tubule Drug Transporters. Clin. J. Am. Soc. Nephrol..

[10] George B., You D., Joy M.S., Aleksunes L.M. (2017). Xenobiotic transporters and kidney injury. Adv. Drug Deliv. Rev..

[11] Risso M.A., Sallustio S., Sueiro V., Bertoni V., Gonzalez-Torres H., Musso C.G. (2019). The Importance of Tubular Function in Chronic Kidney Disease. Int. J. Nephrol. Renov. Dis..

[12] Kalogeris T., Baines C.P., Krenz M., Korthuis R.J. (2012). Cell biology of ischemia/reperfusion injury. Int. Rev. Cell Mol. Biol..

[13] Sheridan A.M., Bonventre J.V. (2000). Cell biology and molecular mechanisms of injury in ischemic acute renal failure. Curr. Opin. Nephrol. Hypertens..

[14] Masereeuw R., Russel F.G.M. (2012). Regulatory pathways for ATP-binding cassette transport proteins in kidney proximal tubules. AAPS J..

[15] Skorecki K., Chertow G., Marsden P., Taal M., Yu A. (2015). Brenner and Rector’s The Kidney, (2 Volume Set).

[16] El-Sheikh A.A.K., Masereeuw R., Russel F.G.M. (2008). Mechanisms of renal anionic drug transport. Eur. J. Pharmacol..

[17] Otani N., Ouchi M., Hayashi K., Jutabha P., Anzai N. (2017). Roles of organic anion transporters (OATs) in renal proximal tubules and their localization. Anat. Sci. Int..

[18] Yin J., Wang J. (2016). Renal drug transporters and their significance in drug–drug interactions. Acta Pharm. Sin. B.

[19] Ekaratanawong S., Anzai N., Jutabha P., Miyazaki H., Noshiro R., Takeda M., Kanai Y., Sophasan S., Endou H. (2004). Human organic anion transporter 4 is a renal apical organic anion/dicarboxylate exchanger in the proximal tubules. J. Pharmacol. Sci..

[20] Motohashi H., Sakurai Y., Saito H., Masuda S., Urakami Y., Goto M., Fukatsu A., Ogawa O., Inui K. (2002). Gene expression levels and immunolocalization of organic ion transporters in the human kidney. J. Am. Soc. Nephrol..

[21] Koepsell H., Endou H. (2004). The SLC22 drug transporter family. Pflug. Arch..

[22] Koepsell H. (2013). The SLC22 family with transporters of organic cations, anions and zwitterions. Mol. Asp. Med..

[23] Pelis R.M., Wright S.H. (2014). SLC22, SLC44, and SLC47 transporters-organic anion and cation transporters: Molecular and cellular properties. Curr. Top. Membr..

[24] Farrow E.G., White K.E. (2010). Recent Advances in Renal Phosphate Handling. Nat. Rev. Nephrol..

[25] Srikant S., Gaudet R. (2019). Mechanics and pharmacology of substrate selection and transport by eukaryotic ABC exporters. Nat. Struct. Mol. Biol..

[26] Aperia A., Fryckstedt J., Holtbäck U., Belusa R., Cheng X.J., Eklöf A.C., Li D., Wang Z.M., Ohtomo Y. (1996). Cellular mechanisms for bi-directional regulation of tubular sodium reabsorption. Kidney Int..

[27] Masereeuw R., Mutsaers H.A.M., Toyohara T., Abe T., Jhawar S., Sweet D.H., Lowenstein J. (2014). The kidney and uremic toxin removal: Glomerulus or tubule?. Semin. Nephrol..

[28] Wu W., Bush K.T., Nigam S.K. (2017). Key Role for the Organic Anion Transporters, OAT1 and OAT3, in the in vivo Handling of Uremic Toxins and Solutes. Sci. Rep..

[29] Ivanyuk A., Livio F., Biollaz J., Buclin T. (2017). Renal Drug Transporters and Drug Interactions. Clin. Pharm..

[30] Burckhardt G. (2012). Drug transport by Organic Anion Transporters (OATs). Pharmacol. Ther..

[31] Koepsell H. (2020). Organic Cation Transporters in Health and Disease. Pharmacol. Rev..

[32] Sharom F.J. (2011). The P-glycoprotein multidrug transporter. Essays Biochem..

[33] Liu X. (2019). SLC Family Transporters. Adv. Exp. Med. Biol..

[34] Fenton R.A., Poulsen S.B., de la Mora Chavez S., Soleimani M., Dominguez Rieg J.A., Rieg T. (2017). Renal tubular NHE3 is required in the maintenance of water and sodium chloride homeostasis. Kidney Int..

[35] Soleimani M., Burnham C.E. (2000). Physiologic and molecular aspects of the Na+:HCO3− cotransporter in health and disease processes. Kidney Int..

[36] Mercado A., Vázquez N., Song L., Cortés R., Enck A.H., Welch R., Delpire E., Gamba G., Mount D.B. (2005). NH2-terminal heterogeneity in the KCC3 K+-Cl- cotransporter. Am. J. Physiol. Ren. Physiol..

[37] Markovich D. (2011). Physiological roles of renal anion transporters NaS1 and Sat1. Am. J. Physiol. Ren. Physiol..

[38] Ghezzi C., Loo D.D.F., Wright E.M. (2018). Physiology of renal glucose handling via SGLT1, SGLT2 and GLUT2. Diabetologia.

[39] Smith D.E., Clémençon B., Hediger M.A. (2013). Proton-coupled oligopeptide transporter family SLC15: Physiological, pharmacological and pathological implications. Mol. Asp. Med..

[40] Verrey F., Singer D., Ramadan T., Vuille-dit-Bille R.N., Mariotta L., Camargo S.M.R. (2009). Kidney amino acid transport. Pflug. Arch..

[41] Turner R.J. (1986). beta-Amino acid transport across the renal brush-border membrane is coupled to both Na and Cl. J. Biol. Chem..

[42] Park S.Y., Kim J.-K., Kim I.J., Choi B.K., Jung K.Y., Lee S., Park K.J., Chairoungdua A., Kanai Y., Endou H. (2005). Reabsorption of neutral amino acids mediated by amino acid transporter LAT2 and TAT1 in the basolateral membrane of proximal tubule. Arch. Pharm. Res..

[43] Vilches C., Boiadjieva-Knöpfel E., Bodoy S., Camargo S., López de Heredia M., Prat E., Ormazabal A., Artuch R., Zorzano A., Verrey F. (2018). Cooperation of Antiporter LAT2/CD98hc with Uniporter TAT1 for Renal Reabsorption of Neutral Amino Acids. J. Am. Soc. Nephrol..

[44] Mora C., Chillarón J., Calonge M.J., Forgo J., Testar X., Nunes V., Murer H., Zorzano A., Palacín M. (1996). The rBAT gene is responsible for L-cystine uptake via the b0,(+)-like amino acid transport system in a “renal proximal tubular” cell line (OK cells). J. Biol. Chem..

[45] Fernández E., Carrascal M., Rousaud F., Abián J., Zorzano A., Palacín M., Chillarón J. (2002). rBAT-b(0,+)AT heterodimer is the main apical reabsorption system for cystine in the kidney. Am. J. Physiol. Ren. Physiol..

[46] Torrents D., Estévez R., Pineda M., Fernández E., Lloberas J., Shi Y.B., Zorzano A., Palacín M. (1998). Identification and characterization of a membrane protein (y+L amino acid transporter-1) that associates with 4F2hc to encode the amino acid transport activity y+L. A candidate gene for lysinuric protein intolerance. J. Biol. Chem..

[47] Bergeron M.J., Clémençon B., Hediger M.A., Markovich D. (2013). SLC13 family of Na^+^-coupled di- and tri-carboxylate/sulfate transporters. Mol. Asp. Med..

[48] Gopal E., Umapathy N.S., Martin P.M., Ananth S., Gnana-Prakasam J.P., Becker H., Wagner C.A., Ganapathy V., Prasad P.D. (2007). Cloning and functional characterization of human SMCT2 (SLC5A12) and expression pattern of the transporter in kidney. Biochim. Biophys. Acta.

[49] Eraly S.A., Vallon V., Vaughn D.A., Gangoiti J.A., Richter K., Nagle M., Monte J.C., Rieg T., Truong D.M., Long J.M. Decreased Renal Organic Anion Secretion and Plasma Accumulation of Endogenous Organic Anions in OAT1 Knock-out Mice. http://www.jbc.org.

[50] Burckhardt G., Burckhardt B.C. (2011). In vitro and in vivo evidence of the importance of organic anion transporters (OATs) in drug therapy. Handb. Exp. Pharmacol..

[51] Bush K.T., Wu W., Lun C., Nigam S.K. (2017). The drug transporter OAT3 (SLC22A8) and endogenous metabolite communication via the gut-liver-kidney axis. J. Biol. Chem..

[52] Uwai Y., Motohashi H., Tsuji Y., Ueo H., Katsura T., Inui K. (2007). Interaction and transport characteristics of mycophenolic acid and its glucuronide via human organic anion transporters hOAT1 and hOAT3. Biochem. Pharmacol..

[53] Russel F.G.M., Koenderink J.B., Masereeuw R. (2008). Multidrug resistance protein 4 (MRP4/ABCC4): A versatile efflux transporter for drugs and signalling molecules. Trends Pharmacol. Sci..

[54] Van de Water F.M., Masereeuw R., Russel F.G.M. (2005). Function and regulation of multidrug resistance proteins (MRPs) in the renal elimination of organic anions. Drug Metab. Rev..

[55] Liu X. (2019). ABC Family Transporters. Adv. Exp. Med. Biol..

[56] Mutsaers H.A.M., Caetano-Pinto P., Seegers A.E.M., Dankers A.C.A., van den Broek P.H.H., Wetzels J.F.M., van den Brand J.A.J.G., van den Heuvel L.P., Hoenderop J.G., Wilmer M.J.G. (2015). Proximal tubular efflux transporters involved in renal excretion of p-cresyl sulfate and p-cresyl glucuronide: Implications for chronic kidney disease pathophysiology. Toxicol. In Vitro.

[57] Dehghan A., Köttgen A., Yang Q., Hwang S.-J., Kao W.L., Rivadeneira F., Boerwinkle E., Levy D., Hofman A., Astor B.C. (2008). Association of three genetic loci with uric acid concentration and risk of gout: A genome-wide association study. Lancet.

[58] Kimura N., Masuda S., Katsura T., Inui K. (2009). Transport of guanidine compounds by human organic cation transporters, hOCT1 and hOCT2. Biochem. Pharmacol..

[59] Bacher P., Giersiefer S., Bach M., Fork C., Schömig E., Gründemann D. (2009). Substrate discrimination by ergothioneine transporter SLC22A4 and carnitine transporter SLC22A5: Gain-of-function by interchange of selected amino acids. Biochim. Biophys. Acta.

[60] Nies A.T., Damme K., Kruck S., Schaeffeler E., Schwab M. (2016). Structure and function of multidrug and toxin extrusion proteins (MATEs) and their relevance to drug therapy and personalized medicine. Arch. Toxicol..

[61] Sharfuddin A.A., Molitoris B.A. (2011). Pathophysiology of ischemic acute kidney injury. Nat. Rev. Nephrol..

[62] Basile D.P., Anderson M.D., Sutton T.A. (2012). Pathophysiology of Acute Kidney Injury. Compr. Physiol..

[63] Guellec C.B.-L., Largeau B., Bon D., Marquet P., Hauet T. (2018). Ischemia/reperfusion-associated tubular cells injury in renal transplantation: Can metabolomics inform about mechanisms and help identify new therapeutic targets?. Pharmacol. Res..

[64] Du J., Zhang L., Yang Y., Li W., Chen L., Ge Y., Sun C., Zhu Y., Gu L. (2010). ATP depletion-induced actin rearrangement reduces cell adhesion via p38 MAPK-HSP27 signaling in renal proximal tubule cells. Cell. Physiol. Biochem..

[65] Molitoris B.A., Dahl R., Geerdes A. (1992). Cytoskeleton disruption and apical redistribution of proximal tubule Na(+)-K(+)-ATPase during ischemia. Am. J. Physiol..

[66] Khundmiri S.J., Asghar M., Khan F., Salim S., Yusufi A.N. (1997). Effect of reversible and irreversible ischemia on marker enzymes of BBM from renal cortical PT subpopulations. Am. J. Physiol..

[67] Collard C.D., Gelman S. (2001). Pathophysiology, clinical manifestations, and prevention of ischemia-reperfusion injury. Anesthesiology.

[68] Pochineni V., Rondon-Berrios H. (2018). Electrolyte and Acid-Base Disorders in the Renal Transplant Recipient. Front. Med. (Lausanne).

[69] Khundmiri S.J., Asghar M., Banday A.A., Khan F., Salim S., Levi M., Yusufi A.N.K. (2005). Effect of ischemia reperfusion on sodium-dependent phosphate transport in renal brush border membranes. Biochim. Biophys. Acta.

[70] Di Sole F., Hu M.-C., Zhang J., Babich V., Bobulescu I.A., Shi M., McLeroy P., Rogers T.E., Moe O.W. (2011). The reduction of Na/H exchanger-3 protein and transcript expression in acute ischemia-reperfusion injury is mediated by extractable tissue factor(s). Kidney Int..

[71] Kwon T.H., Frøkiaer J., Han J.S., Knepper M.A., Nielsen S. (2000). Decreased abundance of major Na(+) transporters in kidneys of rats with ischemia-induced acute renal failure. Am. J. Physiol. Ren. Physiol..

[72] Johnston P.A., Rennke H., Levinsky N.G. (1984). Recovery of proximal tubular function from ischemic injury. Am. J. Physiol..

[73] Molitoris B.A., Kinne R. (1987). Ischemia induces surface membrane dysfunction. Mechanism of altered Na+-dependent glucose transport. J. Clin. Investig..

[74] Saito H. (2010). Pathophysiological regulation of renal SLC22A organic ion transporters in acute kidney injury: Pharmacological and toxicological implications. Pharmacol. Ther..

[75] Bischoff A., Bucher M., Gekle M., Sauvant C. (2014). PAH clearance after renal ischemia and reperfusion is a function of impaired expression of basolateral Oat1 and Oat3. Physiol. Rep..

[76] Schneider R., Sauvant C., Betz B., Otremba M., Fischer D., Holzinger H., Wanner C., Galle J., Gekle M. (2007). Downregulation of organic anion transporters OAT1 and OAT3 correlates with impaired secretion of para-aminohippurate after ischemic acute renal failure in rats. Am. J. Physiol. Ren. Physiol..

[77] Matsuzaki T., Watanabe H., Yoshitome K., Morisaki T., Hamada A., Nonoguchi H., Kohda Y., Tomita K., Inui K., Saito H. (2007). Downregulation of organic anion transporters in rat kidney under ischemia/reperfusion-induced acute [corrected] renal failure. Kidney Int..

[78] Sauvant C., Schneider R., Holzinger H., Renker S., Wanner C., Gekle M. (2009). Implementation of an in vitro model system for investigation of reperfusion damage after renal ischemia. Cell. Physiol. Biochem..

[79] Matsuzaki T., Morisaki T., Sugimoto W., Yokoo K., Sato D., Nonoguchi H., Tomita K., Terada T., Inui K., Hamada A. (2008). Altered pharmacokinetics of cationic drugs caused by down-regulation of renal rat organic cation transporter 2 (Slc22a2) and rat multidrug and toxin extrusion 1 (Slc47a1) in ischemia/reperfusion-induced acute kidney injury. Drug Metab. Dispos..

[80] Schneider R., Meusel M., Betz B., Kersten M., Möller-Ehrlich K., Wanner C., Koepsell H., Sauvant C. (2011). Nitric oxide-induced regulation of renal organic cation transport after renal ischemia-reperfusion injury. Am. J. Physiol. Ren. Physiol..

[81] Ciarimboli G., Schröter R., Neugebauer U., Vollenbröker B., Gabriëls G., Brzica H., Sabolić I., Pietig G., Pavenstädt H., Schlatter E. (2013). Kidney transplantation down-regulates expression of organic cation transporters, which translocate β-blockers and fluoroquinolones. Mol. Pharm..

[82] Corrigan G., Ramaswamy D., Kwon O., Sommer F.G., Alfrey E.J., Dafoe D.C., Olshen R.A., Scandling J.D., Myers B.D. (1999). PAH extraction and estimation of plasma flow in human postischemic acute renal failure. Am. J. Physiol..

[83] Kwon O., Hong S.-M., Blouch K. (2007). Alteration in renal organic anion transporter 1 after ischemia/reperfusion in cadaveric renal allografts. J. Histochem. Cytochem..

[84] Huls M., van den Heuvel J.J.M.W., Dijkman H.B.P.M., Russel F.G.M., Masereeuw R. (2006). ABC transporter expression profiling after ischemic reperfusion injury in mouse kidney. Kidney Int..

[85] Schneider R., Meusel M., Betz B., Held C., Möller-Ehrlich K., Büttner-Herold M., Wanner C., Gekle M., Sauvant C. (2015). Oat1/3 restoration protects against renal damage after ischemic AKI. Am. J. Physiol. Ren. Physiol..

[86] Hagos Y., Schley G., Schödel J., Krick W., Burckhardt G., Willam C., Burckhardt B.C. (2012). α-Ketoglutarate-related inhibitors of HIF prolyl hydroxylases are substrates of renal organic anion transporters 1 (OAT1) and 4 (OAT4). Pflug. Arch..

[87] Paulusma C.C., Kothe M.J., Bakker C.T., Bosma P.J., van Bokhoven I., van Marle J., Bolder U., Tytgat G.N., Oude Elferink R.P. (2000). Zonal down-regulation and redistribution of the multidrug resistance protein 2 during bile duct ligation in rat liver. Hepatology.

[88] Ke Q., Costa M. (2006). Hypoxia-inducible factor-1 (HIF-1). Mol. Pharmacol..

[89] Hill P., Shukla D., Tran M.G.B., Aragones J., Cook H.T., Carmeliet P., Maxwell P.H. (2008). Inhibition of hypoxia inducible factor hydroxylases protects against renal ischemia-reperfusion injury. J. Am. Soc. Nephrol..

[90] Conde E., Alegre L., Blanco-Sánchez I., Sáenz-Morales D., Aguado-Fraile E., Ponte B., Ramos E., Sáiz A., Jiménez C., Ordoñez A. (2012). Hypoxia inducible factor 1-alpha (HIF-1 alpha) is induced during reperfusion after renal ischemia and is critical for proximal tubule cell survival. PLoS ONE.

[91] Chen Y., Jiang S., Zou J., Zhong Y., Ding X. (2016). Silencing HIF-1α aggravates growth inhibition and necrosis of proximal renal tubular epithelial cell under hypoxia. Ren. Fail..

[92] Comerford K.M., Wallace T.J., Karhausen J., Louis N.A., Montalto M.C., Colgan S.P. (2002). Hypoxia-inducible factor-1-dependent regulation of the multidrug resistance (MDR1) gene. Cancer Res..

[93] Zapata-Morales J.R., Galicia-Cruz O.G., Franco M., Martinez Y., Morales F. (2014). Hypoxia-inducible factor-1α (HIF-1α) protein diminishes sodium glucose transport 1 (SGLT1) and SGLT2 protein expression in renal epithelial tubular cells (LLC-PK1) under hypoxia. J. Biol. Chem..

[94] Jiang W., Prokopenko O., Wong L., Inouye M., Mirochnitchenko O. (2005). IRIP, a new ischemia/reperfusion-inducible protein that participates in the regulation of transporter activity. Mol. Cell. Biol..

[95] Li Q., Yang H., Peng X., Guo D., Dong Z., Polli J.E., Shu Y. (2013). Ischemia/Reperfusion-inducible protein modulates the function of organic cation transporter 1 and multidrug and toxin extrusion 1. Mol. Pharm..

[96] Prokopenko O., Mirochnitchenko O. (2009). Ischemia-reperfusion-inducible protein modulates cell sensitivity to anticancer drugs by regulating activity of efflux transporter. Am. J. Physiol. Cell Physiol..

[97] Kwon O., Wang W.-W., Miller S. (2008). Renal organic anion transporter 1 is maldistributed and diminishes in proximal tubule cells but increases in vasculature after ischemia and reperfusion. Am. J. Physiol. Ren. Physiol..

[98] Wolff N.A., Thies K., Kuhnke N., Reid G., Friedrich B., Lang F., Burckhardt G. (2003). Protein kinase C activation downregulates human organic anion transporter 1-mediated transport through carrier internalization. J. Am. Soc. Nephrol..

[99] Preising C., Schneider R., Bucher M., Gekle M., Sauvant C. (2015). Regulation of Expression of Renal Organic Anion Transporters OAT1 and OAT3 in a Model of Ischemia/Reperfusion Injury. Cell. Physiol. Biochem..

[100] Malagrino P.A., Venturini G., Yogi P.S., Dariolli R., Padilha K., Kiers B., Gois T.C., Motta-Leal-Filho J.M., Takimura C.K., Girardi A.C.C. (2016). Metabolomic characterization of renal ischemia and reperfusion in a swine model. Life Sci..

[101] Wei Q., Xiao X., Fogle P., Dong Z. (2014). Changes in metabolic profiles during acute kidney injury and recovery following ischemia/reperfusion. PLoS ONE.

[102] Jouret F., Leenders J., Poma L., Defraigne J.-O., Krzesinski J.-M., de Tullio P. (2016). Nuclear Magnetic Resonance Metabolomic Profiling of Mouse Kidney, Urine and Serum Following Renal Ischemia/Reperfusion Injury. PLoS ONE.

[103] Chihanga T., Ma Q., Nicholson J.D., Ruby H.N., Edelmann R.E., Devarajan P., Kennedy M.A. (2018). NMR spectroscopy and electron microscopy identification of metabolic and ultrastructural changes to the kidney following ischemia-reperfusion injury. Am. J. Physiol. Ren. Physiol..

[104] Bon D., Billault C., Claire B., Thuillier R., Hebrard W., Boildieu N., Celhay O., Irani J., Seguin F., Hauet T. (2014). Analysis of perfusates during hypothermic machine perfusion by NMR spectroscopy: A potential tool for predicting kidney graft outcome. Transplantation.

[105] Guy A.J., Nath J., Cobbold M., Ludwig C., Tennant D.A., Inston N.G., Ready A.R. (2015). Metabolomic analysis of perfusate during hypothermic machine perfusion of human cadaveric kidneys. Transplantation.

[106] Stryjak I., Warmuzińska N., Bogusiewicz J., Łuczykowski K., Bojko B. (2020). Monitoring of the influence of long-term oxidative stress and ischemia on the condition of kidney using solid phase microextraction chemical biopsy coupled with liquid chromatography high resolution mass spectrometry. J. Sep. Sci..

[107] Nath J., Smith T.B., Patel K., Ebbs S.R., Hollis A., Tennant D.A., Ludwig C., Ready A.R. (2017). Metabolic differences between cold stored and machine perfused porcine kidneys: A 1H NMR based study. Cryobiology.

[108] DiRito J.R., Hosgood S.A., Tietjen G.T., Nicholson M.L. (2018). The future of marginal kidney repair in the context of normothermic machine perfusion. Am. J. Transplant..

[109] Weissenbacher A., Vrakas G., Nasralla D., Ceresa C.D.L. (2019). The future of organ perfusion and re-conditioning. Transpl. Int..

[110] Woroniecki R., Ferdinand J.R., Morrow J.S., Devarajan P. (2003). Dissociation of spectrin-ankyrin complex as a basis for loss of Na-K-ATPase polarity after ischemia. Am. J. Physiol. Ren. Physiol..

[111] Fernández-Llama P., Andrews P., Turner R., Saggi S., Dimari J., Kwon T.H., Nielsen S., Safirstein R., Knepper M.A. (1999). Decreased abundance of collecting duct aquaporins in post-ischemic renal failure in rats. J. Am. Soc. Nephrol..

[112] Miltényi M., Tulassay T., Körner A., Szabó A., Dobos M. (1988). Tubular dysfunction in metabolic acidosis. First step to acute renal failure. Contrib. Nephrol..

[113] Liu J., Litt L., Segal M.R., Kelly M.J.S., Pelton J.G., Kim M. (2011). Metabolomics of oxidative stress in recent studies of endogenous and exogenously administered intermediate metabolites. Int. J. Mol. Sci..

[114] Halliwell B., Cheah I.K., Drum C.L. (2016). Ergothioneine, an adaptive antioxidant for the protection of injured tissues? A hypothesis. Biochem. Biophys. Res. Commun..

[115] Mishra A., Reddy I.J., Gupta P.S.P., Mondal S. (2016). L-carnitine Mediated Reduction in Oxidative Stress and Alteration in Transcript Level of Antioxidant Enzymes in Sheep Embryos Produced In Vitro. Reprod. Domest. Anim..

[116] Ribas G.S., Vargas C.R., Wajner M. (2014). L-carnitine supplementation as a potential antioxidant therapy for inherited neurometabolic disorders. Gene.

[117] Liu Y., Yan S., Ji C., Dai W., Hu W., Zhang W., Mei C. (2012). Metabolomic changes and protective effect of (L)-carnitine in rat kidney ischemia/reperfusion injury. Kidney Blood Press. Res..

[118] Chouchani E.T., Pell V.R., Gaude E., Aksentijević D., Sundier S.Y., Robb E.L., Logan A., Nadtochiy S.M., Ord E.N.J., Smith A.C. (2014). Ischaemic accumulation of succinate controls reperfusion injury through mitochondrial ROS. Nature.

[119] Chinopoulos C. (2013). Which way does the citric acid cycle turn during hypoxia? The critical role of α-ketoglutarate dehydrogenase complex. J. Neurosci. Res..

[120] Tajima T., Yoshifuji A., Matsui A., Itoh T., Uchiyama K., Kanda T., Tokuyama H., Wakino S., Itoh H. (2019). β-hydroxybutyrate attenuates renal ischemia-reperfusion injury through its anti-pyroptotic effects. Kidney Int..

[121] Ferenbach D.A., Bonventre J.V. (2015). Mechanisms of maladaptive repair after AKI leading to accelerated kidney ageing and CKD. Nat. Rev. Nephrol..

[122] Andrade L., Rodrigues C.E., Gomes S.A., Noronha I.L. (2018). Acute Kidney Injury as a Condition of Renal Senescence. Cell Transplant..

[123] Qi R., Yang C. (2018). Renal tubular epithelial cells: The neglected mediator of tubulointerstitial fibrosis after injury. Cell Death Dis..

[124] Huls M., Russel F.G.M., Masereeuw R. (2009). The Role of ATP Binding Cassette Transporters in Tissue Defense and Organ Regeneration. J. Pharm. Exp. Ther..

[125] Humphreys B.D., Czerniak S., DiRocco D.P., Hasnain W., Cheema R., Bonventre J.V. (2011). Repair of injured proximal tubule does not involve specialized progenitors. Proc. Natl. Acad. Sci. USA.

[126] Berger K., Moeller M.J. (2014). Mechanisms of epithelial repair and regeneration after acute kidney injury. Semin. Nephrol..

[127] Ahn S.-Y., Nigam S.K. (2009). Toward a systems level understanding of organic anion and other multispecific drug transporters: A remote sensing and signaling hypothesis. Mol. Pharmacol. Exp..

[128] Rosenthal S.B., Bush K.T., Nigam S.K. (2019). A Network of SLC and ABC Transporter and DME Genes Involved in Remote Sensing and Signaling in the Gut-Liver-Kidney Axis. Sci. Rep..

[129] Nigam S.K., Bush K.T. (2019). Uraemic syndrome of chronic kidney disease: Altered remote sensing and signalling. Nat. Rev. Nephrol..

[130] Chen C., Slitt A.L., Dieter M.Z., Tanaka Y., Scheffer G.L., Klaassen C.D. (2005). Up-regulation of Mrp4 expression in kidney of Mrp2-deficient TR- rats. Biochem. Pharmacol..

[131] Shiao C.-C., Wu P.-C., Huang T.-M., Lai T.-S., Yang W.-S., Wu C.-H., Lai C.-F., Wu V.-C., Chu T.-S., Wu K.-D. (2015). Long-term remote organ consequences following acute kidney injury. Crit. Care.

[132] Dépret F., Prud’homme M., Legrand M. (2017). A Role of Remote Organs Effect in Acute Kidney Injury Outcome. Nephron.

[133] Kao C.-C., Yang W.-S., Fang J.-T., Liu K.D., Wu V.-C. (2019). Remote organ failure in acute kidney injury. J. Formos. Med. Assoc..

[134] Serteser M., Koken T., Kahraman A., Yilmaz K., Akbulut G., Dilek O.N. (2002). Changes in hepatic TNF-alpha levels, antioxidant status, and oxidation products after renal ischemia/reperfusion injury in mice. J. Surg. Res..

[135] Vaghasiya J.D., Sheth N.R., Bhalodia Y.S., Jivani N.P. (2010). Exaggerated Liver Injury Induced by Renal Ischemia Reperfusion in Diabetes: Effect of Exenatide. Saudi J. Gastroenterol..

[136] Dou L., Bertrand E., Cerini C., Faure V., Sampol J., Vanholder R., Berland Y., Brunet P. (2004). The uremic solutes p-cresol and indoxyl sulfate inhibit endothelial proliferation and wound repair. Kidney Int..

[137] Dou L., Jourde-Chiche N., Faure V., Cerini C., Berland Y., Dignat-George F., Brunet P. (2007). The uremic solute indoxyl sulfate induces oxidative stress in endothelial cells. J. Thromb. Haemost..

[138] Huang M., Wei R., Wang Y., Su T., Li P., Chen X. (2018). The uremic toxin hippurate promotes endothelial dysfunction via the activation of Drp1-mediated mitochondrial fission. Redox Biol..

[139] Brandoni A., Torres A.M. (2015). Altered Renal Expression of Relevant Clinical Drug Transporters in Different Models of Acute Uremia in Rats. Role of Urea Levels. Cell. Physiol. Biochem..

[140] Knoflach A., Binswanger U. (1994). Serum hippuric acid concentration in renal allograft rejection, ureter obstruction, and tubular necrosis. Transpl. Int..

[141] Satoh M., Hayashi H., Watanabe M., Ueda K., Yamato H., Yoshioka T., Motojima M. (2003). Uremic toxins overload accelerates renal damage in a rat model of chronic renal failure. Nephron Exp. Nephrol..

[142] Oliva-Damaso E., Oliva-Damaso N., Rodriguez-Esparragon F., Payan J., Baamonde-Laborda E., Gonzalez-Cabrera F., Santana-Estupiñan R., Rodriguez-Perez J.C. (2019). Asymmetric (ADMA) and Symmetric (SDMA) Dimethylarginines in Chronic Kidney Disease: A Clinical Approach. Int. J. Mol. Sci..

[143] Gondouin B., Cerini C., Dou L., Sallée M., Duval-Sabatier A., Pletinck A., Calaf R., Lacroix R., Jourde-Chiche N., Poitevin S. (2013). Indolic uremic solutes increase tissue factor production in endothelial cells by the aryl hydrocarbon receptor pathway. Kidney Int..

[144] Barreto F.C., Barreto D.V., Canziani M.E.F. (2017). Uremia Retention Molecules and Clinical Outcomes. Contrib Nephrol..

[145] Liabeuf S., Cheddani L., Massy Z.A. (2018). Uremic Toxins and Clinical Outcomes: The Impact of Kidney Transplantation. Toxins (Basel).

[146] Massy Z.A., Liabeuf S. (2017). Middle-Molecule Uremic Toxins and Outcomes in Chronic Kidney Disease. Contrib. Nephrol..

[147] Mao Q., Unadkat J.D. (2015). Role of the breast cancer resistance protein (BCRP/ABCG2) in drug transport-an update. AAPS J..

[148] Patel C.G., Ogasawara K., Akhlaghi F. (2013). Mycophenolic acid glucuronide is transported by multidrug resistance-associated protein 2 and this transport is not inhibited by cyclosporine, tacrolimus or sirolimus. Xenobiotica.

[149] Naesens M., Lerut E., de Jonge H., Van Damme B., Vanrenterghem Y., Kuypers D.R.J. (2009). Donor age and renal P-glycoprotein expression associate with chronic histological damage in renal allografts. J. Am. Soc. Nephrol..

[150] Hauser I.A., Schaeffeler E., Gauer S., Scheuermann E.H., Wegner B., Gossmann J., Ackermann H., Seidl C., Hocher B., Zanger U.M. (2005). ABCB1 genotype of the donor but not of the recipient is a major risk factor for cyclosporine-related nephrotoxicity after renal transplantation. J. Am. Soc. Nephrol..

[151] Moore J., McKnight A.J., Döhler B., Simmonds M.J., Courtney A.E., Brand O.J., Briggs D., Ball S., Cockwell P., Patterson C.C. (2012). Donor ABCB1 variant associates with increased risk for kidney allograft failure. J. Am. Soc. Nephrol..

[152] Woillard J.-B., Gatault P., Picard N., Arnion H., Anglicheau D., Marquet P. (2018). A donor and recipient candidate gene association study of allograft loss in renal transplant recipients receiving a tacrolimus-based regimen. Am. J. Transplant..

[153] Kimchi-Sarfaty C., Oh J.M., Kim I.-W., Sauna Z.E., Calcagno A.M., Ambudkar S.V., Gottesman M.M. (2007). A “silent” polymorphism in the MDR1 gene changes substrate specificity. Science.

[154] El-Sheikh A.A.K., Greupink R., Wortelboer H.M., van den Heuvel J.J.M.W., Schreurs M., Koenderink J.B., Masereeuw R., Russel F.G.M. (2013). Interaction of immunosuppressive drugs with human organic anion transporter (OAT) 1 and OAT3, and multidrug resistance-associated protein (MRP) 2 and MRP4. Transl Res..

[155] Nies A.T., Cantz T., Brom M., Leier I., Keppler D. (1998). Expression of the apical conjugate export pump, Mrp2, in the polarized hepatoma cell line, WIF-B. Hepatology.

[156] Zimmerman J.J., Harper D., Getsy J., Jusko W.J. (2003). Pharmacokinetic interactions between sirolimus and microemulsion cyclosporine when orally administered jointly and 4 hours apart in healthy volunteers. J. Clin. Pharm..

[157] Emoto C., Vinks A.A., Fukuda T. (2016). Risk Assessment of Drug-Drug Interactions of Calcineurin Inhibitors Affecting Sirolimus Pharmacokinetics in Renal Transplant Patients. Ther. Drug Monit..

[158] Podder H., Stepkowski S.M., Napoli K.L., Clark J., Verani R.R., Chou T.C., Kahan B.D. (2001). Pharmacokinetic interactions augment toxicities of sirolimus/cyclosporine combinations. J. Am. Soc. Nephrol..

[159] Kahan B.D. (2000). Efficacy of sirolimus compared with azathioprine for reduction of acute renal allograft rejection: A randomised multicentre study. The Rapamune US Study Group. Lancet.

[160] Anglicheau D., Pallet N., Rabant M., Marquet P., Cassinat B., Méria P., Beaune P., Legendre C., Thervet E. (2006). Role of P-glycoprotein in cyclosporine cytotoxicity in the cyclosporine-sirolimus interaction. Kidney Int..

[161] Hesselink D.A., van Hest R.M., Mathot R.A.A., Bonthuis F., Weimar W., de Bruin R.W.F., van Gelder T. (2005). Cyclosporine interacts with mycophenolic acid by inhibiting the multidrug resistance-associated protein 2. Am. J. Transplant..

[162] El-Sheikh A.A.K., Koenderink J.B., Wouterse A.C., van den Broek P.H.H., Verweij V.G.M., Masereeuw R., Russel F.G.M. (2014). Renal glucuronidation and multidrug resistance protein 2-/ multidrug resistance protein 4-mediated efflux of mycophenolic acid: Interaction with cyclosporine and tacrolimus. Transl. Res..

[163] Nigam S.K. (2015). What do drug transporters really do?. Nat. Rev. Drug Discov.

[164] Sakurai Y., Motohashi H., Ueo H., Masuda S., Saito H., Okuda M., Mori N., Matsuura M., Doi T., Fukatsu A. (2004). Expression levels of renal organic anion transporters (OATs) and their correlation with anionic drug excretion in patients with renal diseases. Pharm. Res..

[165] Kito T., Ito S., Mizuno T., Maeda K., Kusuhara H. (2019). Investigation of non-linear Mate1-mediated efflux of trimethoprim in the mouse kidney as the mechanism underlying drug-drug interactions between trimethoprim and organic cations in the kidney. Drug Metab. Pharmacol..

[166] Saat T.C., van den Akker E.K., IJzermans J.N.M., Dor F.J.M.F., de Bruin R.W.F. (2016). Improving the outcome of kidney transplantation by ameliorating renal ischemia reperfusion injury: Lost in translation?. J. Transl. Med..

[167] Nicholson M.L., Hosgood S.A. (2013). Renal transplantation after ex vivo normothermic perfusion: The first clinical study. Am. J. Transplant..

[168] Kaths J.M., Cen J.Y., Chun Y.M., Echeverri J., Linares I., Ganesh S., Yip P., John R., Bagli D., Mucsi I. (2017). Continuous Normothermic Ex Vivo Kidney Perfusion Is Superior to Brief Normothermic Perfusion Following Static Cold Storage in Donation After Circulatory Death Pig Kidney Transplantation. Am. J. Transplant..

[169] Kataria A., Magoon S., Makkar B., Gundroo A. (2019). Machine perfusion in kidney transplantation. Curr. Opin. Organ Transplant..

[170] Chatterjee P.K. (2007). Novel pharmacological approaches to the treatment of renal ischemia-reperfusion injury: A comprehensive review. Naunyn Schmiedebergs Arch. Pharmacol..

[171] Chatauret N., Thuillier R., Hauet T. (2011). Preservation strategies to reduce ischemic injury in kidney transplantation: Pharmacological and genetic approaches. Curr. Opin. Organ Transplant..

[172] Gong H., Sun J., Xue W., Tian P., Ding X., Yan H., Li Y., Zheng J. (2014). Protective effect of truncated Na+/K+-ATPase β on ischemia/reperfusion-induced renal injury in rats. Exp. Biol. Med. (Maywood).

[173] Belliard A., Gulati G.K., Duan Q., Alves R., Brewer S., Madan N., Sottejeau Y., Wang X., Kalisz J., Pierre S.V. (2016). Ischemia/reperfusion-induced alterations of enzymatic and signaling functions of the rat cardiac Na+/K+-ATPase: Protection by ouabain preconditioning. Physiol. Rep..

[174] Schneider R., Meusel M., Renker S., Bauer C., Holzinger H., Roeder M., Wanner C., Gekle M., Sauvant C. (2009). Low-dose indomethacin after ischemic acute kidney injury prevents downregulation of Oat1/3 and improves renal outcome. Am. J. Physiol. Ren. Physiol..

[175] Saigo C., Nomura Y., Yamamoto Y., Sagata M., Matsunaga R., Jono H., Nishi K., Saito H. (2014). Meclofenamate elicits a nephropreventing effect in a rat model of ischemic acute kidney injury by suppressing indoxyl sulfate production and restoring renal organic anion transporters. Drug Des. Dev. Ther..

[176] Verma R., Mishra V., Sasmal D., Raghubir R. (2010). Pharmacological evaluation of glutamate transporter 1 (GLT-1) mediated neuroprotection following cerebral ischemia/reperfusion injury. Eur. J. Pharmacol..

[177] Yamashita J., Ohkita M., Takaoka M., Kaneshiro Y., Matsuo T., Kaneko K., Matsumura Y. (2007). Role of Na+/H+ exchanger in the pathogenesis of ischemic acute renal failure in mice. J. Cardiovasc. Pharmacol..

[178] Hropot M., Juretschke H.P., Langer K.H., Schwark J.R. (2001). S3226, a novel NHE3 inhibitor, attenuates ischemia-induced acute renal failure in rats. Kidney Int..

[179] Liu W.-H., Liu H.-B., Gao D.-K., Ge G.-Q., Zhang P., Sun S.-R., Wang H.-M., Liu S.-B. (2013). ABCG2 protects kidney side population cells from hypoxia/reoxygenation injury through activation of the MEK/ERK pathway. Cell Transplant..

[180] Kieran N.E., Doran P.P., Connolly S.B., Greenan M.-C., Higgins D.F., Leonard M., Godson C., Taylor C.T., Henger A., Kretzler M. (2003). Modification of the transcriptomic response to renal ischemia/reperfusion injury by lipoxin analog. Kidney Int..

[181] Toyohara T., Suzuki T., Morimoto R., Akiyama Y., Souma T., Shiwaku H.O., Takeuchi Y., Mishima E., Abe M., Tanemoto M. (2009). SLCO4C1 transporter eliminates uremic toxins and attenuates hypertension and renal inflammation. J. Am. Soc. Nephrol..

[182] Nespoux J., Patel R., Hudkins K.L., Huang W., Freeman B., Kim Y.C., Koepsell H., Alpers C.E., Vallon V. (2019). Gene deletion of the Na+-glucose cotransporter SGLT1 ameliorates kidney recovery in a murine model of acute kidney injury induced by ischemia-reperfusion. Am. J. Physiol. Ren. Physiol..

[183] Fuller T.F., Kusch A., Chaykovska L., Catar R., Pützer J., Haller M., Troppmair J., Hoff U., Dragun D. (2012). Protein kinase C inhibition ameliorates posttransplantation preservation injury in rat renal transplants. Transplantation.

[184] Nangaku M., Rosenberger C., Heyman S.N., Eckardt K.-U. (2013). Regulation of hypoxia-inducible factor in kidney disease. Clin. Exp. Pharmacol. Physiol..

[185] Shu S., Wang Y., Zheng M., Liu Z., Cai J., Tang C., Dong Z. (2019). Hypoxia and Hypoxia-Inducible Factors in Kidney Injury and Repair. Cells.

[186] Eltzschig H.K., Eckle T. (2011). Ischemia and reperfusion—From mechanism to translation. Nat. Med..

